# Tandem RNA Chimeras Contribute to Transcriptome Diversity in Human Population and Are Associated with Intronic Genetic Variants

**DOI:** 10.1371/journal.pone.0104567

**Published:** 2014-08-18

**Authors:** Liliana Greger, Jing Su, Johan Rung, Pedro G. Ferreira, Geuvadis consortium, Tuuli Lappalainen, Emmanouil T. Dermitzakis, Alvis Brazma

**Affiliations:** 1 European Molecular Biology Laboratory - European Bioinformatics Institute (EMBL-EBI), Hinxton, United Kingdom; 2 Department of Genetic Medicine and Development, University of Geneva Medical School, Geneva, Switzerland; 3 Institute for Genetics and Genomics in Geneva (iG3), University of Geneva, Geneva, Switzerland; 4 Swiss Institute of Bioinformatics, Geneva, Switzerland; 5 New York Genome Center, New York, New York, United States of America; The John Curtin School of Medical Research, Australia

## Abstract

Chimeric RNAs originating from two or more different genes are known to exist not only in cancer, but also in normal tissues, where they can play a role in human evolution. However, the exact mechanism of their formation is unknown. Here, we use RNA sequencing data from 462 healthy individuals representing 5 human populations to systematically identify and in depth characterize 81 RNA tandem chimeric transcripts, 13 of which are novel. We observe that 6 out of these 81 chimeras have been regarded as cancer-specific. Moreover, we show that a prevalence of long introns at the fusion breakpoint is associated with the chimeric transcripts formation. We also find that tandem RNA chimeras have lower abundances as compared to their partner genes. Finally, by combining our results with genomic data from the same individuals we uncover intronic genetic variants associated with the chimeric RNA formation. Taken together our findings provide an important insight into the chimeric transcripts formation and open new avenues of research into the role of intronic genetic variants in post-transcriptional processing events.

## Introduction

Transcripts formed from exons of two or more different genes are known to play a major role in cancer development [1], where they often arise mostly from gene fusions caused by chromosomal rearrangements. However, chimeric transcripts are also present in healthy human tissues, where they may increase protein complexity and contribute to human evolution [2]–[7]. It is believed that chimeric transcripts in normal population arise from trans-splicing [8] or represent by-passing the transcription termination events [5], [6], [9].

The most prevalent type of chimeric transcripts found in the human genome are tandem RNA chimeras originating from genes residing on the same strand and chromosome [2]–[7]. These RNA chimeras, also known as read-throughs and conjoined genes have the potential for increasing the protein diversity as apparent from reports showing evidence of protein translation in organisms such as avian species [10]. These transcripts are potentially playing an important role in mammalian development, for example in regulating cell development in human [11] and in other organisms such as chick, quail and mouse [10], [12]. Furthermore, many tandem RNA chimeras were found to be evolutionary conserved [13]. Despite the numerous reports showing the existence of these chimeric transcripts in human, the exact mechanism of their formation is still unknown. Kim at all proposed that these events might be caused by removal of the poly (A) signal sequence from the upstream gene region by a deletion or a truncation mutation [14]. Trans-regulator of a poly(A) choice was reported to regulate the chimeric RNA formation in Arabidopsis thaliana [15]. There are also reports of intergenic splicing between tandem genes excluding the possibility of a read-through long transcript [11]. More detailed studies focused on chimeric transcript formation using large datasets of healthy individuals are needed to improve our knowledge of this phenomenon. Most of the reports that studied these events were previously based on ESTs data, which typically included also samples from non-healthy individuals, while at the same time were incomplete representations of the human genome.

Here, for a first time we identified and characterized chimeric transcripts found in a large cohort of population sample of individuals lacking severe phenotypes by using RNA sequencing data isolated from lymphoblastoid cell lines of 462 individuals representing 5 populations from the 1000 Genomes project. By taking advantage of the genomic data available from the 1000 Genomes project, we could link for a first time the mechanism of tandem RNA chimera formation to specific genetic variants.

## Materials and Methods

### Chimeric RNAs discovery

In this study we used RNA sequencing data from lymphoblastoid cell lines of 462 individuals from five populations 91 CEPH (CEU, Utah residents with ancestry from northern and western Europe), 95 Finns (FIN, Finish in Finland), 94 British (GBR, British in England and Scotland), 93 Toscani (TIS, Toscani in Italia) and 89 Yoruba (YRI, Yoruba in Ibadan, Nigeria), who participated in the 1000 Genomes project (study design described in [16]. Chimeric transcripts were identified with a pipeline based on FusionMap v 2013-02-01 [17]. In essence, the raw sequence reads were aligned with bwa aligner [18]. Subsequently, the unmapped reads were extracted from the bam files with SAMtools using the flags for unmapped reads, [19] and then converted to fastq files with the Hydra package [20], which were subsequently subjected to fusion genes analysis with FusionMap [17]. The list of fusion genes was further filtered by the number of split reads supporting the fusion (>2). Finally, to reduce the number of false positive results chimeras, we used RepeatMasker (http://www.repeatmasker.org) to filter out breakpoints overlapping with repetitive elements.

To ensure that the observed population specific fusions were not due to the lack of expression of the partner genes in the other populations, only genes with positive RPKM values observed in all populations were considered. The expression values (RPKM) were estimated with Flux Capacitor v 1.2.3 [21].

The calculation of the open reading frame of the fusions was based on the CDS information in the gene model, provided by the reference GTF file and it is implemented in the FusionMap software.

Finally, the novelty of the fusions was assessed via queries against known annotation databases for read-through events: ConjoinG [7], AceView [22] and literature search.

The distances between the fusion breakpoints was estimated as |bp2 – bp1|, where bp1 is the breakpoint of upstream parent gene and bp2 is the breakpoint of the downstream parent gene involved in the RNA chimera. The frequency counts for each chimeric transcript were estimated as the number of individuals from each population harboring the chimeric transcript.

The sizes of the exons and introns residing at the fusion breakpoint were estimated by taking into account all possible transcript combinations between the chimeric genes.

Distances, frequency plots and intron lengths distribution plot were generated with ggplot2 package within R environment [23].

The exon exclusion pattern was assessed by estimating the frequencies of the excluded exons from the two parent genes during the tandem RNA chimera formation. We took into account all possible transcript combinations.

As a reference annotation for all analyses above we used Gencode v12.

### Quantification of RNA chimeras

We created custom modified Gencode v12 reference gtf file to accommodate all possible transcript isoforms of tandem RNA chimeras by combining exons of the upstream partner gene up to the fusion breakpoint with exons of the downstream partner gene after the fusion breakpoint and assigning a new transcript ID. Then, for each fusion we assigned a new gene ID. In four cases the breakpoint was predicted to be away from the exon-intron boundaries and therefore, we modified the exon boundary to match the predicted breakpoint. Subsequently, all bam files mapped with GEM [24] were subjected to Flux Capacitor, which assigns the reads to the annotated in the custom gtf exon-intron structures [25] to quantify the transcript abundances for each gene. The gene level RPKMs were obtained by summing up the RPKMs for all transcripts sharing the same gene identity. The distribution of the chimeras RPKMs, estimated in individuals harboring the chimeric transcripts was further compared to the RPKMs of the partner genes and all other genes in the same individuals.

All custom scripts and the modified gtf file are available on request.

### Association between tandem RNA chimera formation and genetic variants

The association between tandem RNA chimera formation and genetic variants has been performed on custom annotated 1000 Genomes Phase I, release v3 vcf files, based on Gencode v12 [16] with tools within SnpSift v3.2 toolset [26]. We selected only fusions with frequency higher than 5% in all combined populations and SNPs with MAF greater than 5% with SnpSift filter [26]. Initially, for each fusion we extracted all variants located on both fusion partner genes (+200 nucleotides outside the gene boundaries) with SnpSift Intervals [26]. Then, we tested for association of each fusion with genetic variants in the cases (samples, which have the fusion) compared to the controls (samples, which do not harbor the fusion) by applying Cochran–Armitage test with SnpSift CaseControl [26]. We further selected all variants with adjusted by bonferroni p value<0.05. Subsequently, for each variant, we estimated the OR (odd ratio i.e. proportions of individuals, with the fusion having the alternative allele relative to proportion of individuals without the fusion having the alternative allele).

Further, the identified variants were functionally annotated by utilizing data available from Ensembl regulatory build. Subsequently, the associated with RNA chimeras variants were overlapping with genomic locations for transcripts associated with RNA binding proteins by using data in bed format produced by RIP-chip GeneST, Ribonomic profiling on Affymetrix GeneChip® Human Gene 1.0 ST Arrays [27] as part of the ENCODE project [28]. Overlapping of genomic regions was assessed with bedtools [29]. The identified variants were further inspected with regional association plots performed with LocusZoom v 1.1 [32].

To explore variants in poly(A) sites and signals, we used annotated by Havana, Poly(A) sites and signals Gencode v12 [30] and predicted putative cleavage sites [31]. The prediction of putative cleavage sites was performed by selecting reads containing a poly(A) tail or a poly T head, filtering out low complexity reads, trimming and mapping the remaining reads uniquely to the genome. All 3′ UTR variants overlapping with these sites were inspected further. For each 5′ partner gene we filtered out the observed variants in poly(A) sites or signals by MAF (equal or higher than the gene frequency in the population).

## Results

### Detection and characterization of RNA chimeras

We used a pipeline based on FusionMap software tool [17] to detect fusion genes in RNA sequence data from 462 normal individuals from five different populations. This tool has been proved to be a good compromise in terms of specificity and sensitivity in normal tissues as compared to five other tools for fusion genes discovery [33]. The discovered fusions were classified as intra-chromosomal fusions on the same strand, intra-chromosomal fusions on different strands and inter-chromosomal fusions. For chimeric transcripts arising from intra-chromosomal genes residing on the same strand we used the term “tandem RNA chimeras”.

We observed 89 intrachromosomal genes residing on the same strand, 8 of which implied inversion since the direction of the fusion transcript was inverted relative to the annotation (Table S1 in File S1). In order to study in more detail fusions potentially formed by intergenic splicing than genomic rearrangements, we excluded these 8 fusions from further analysis. (Table S2 in File S1). Except in 10 cases, the breakpoint coincided with the exon boundaries. In 6 partner genes, the fusion breakpoint was located within 1–3 nucleotides from the exon-intron boundary and in 4 cases the breakpoint was predicted to be located inside the exon. Sixty-seven out of the 81 fusions were known, while 13 were novel.

**Figure 1:**
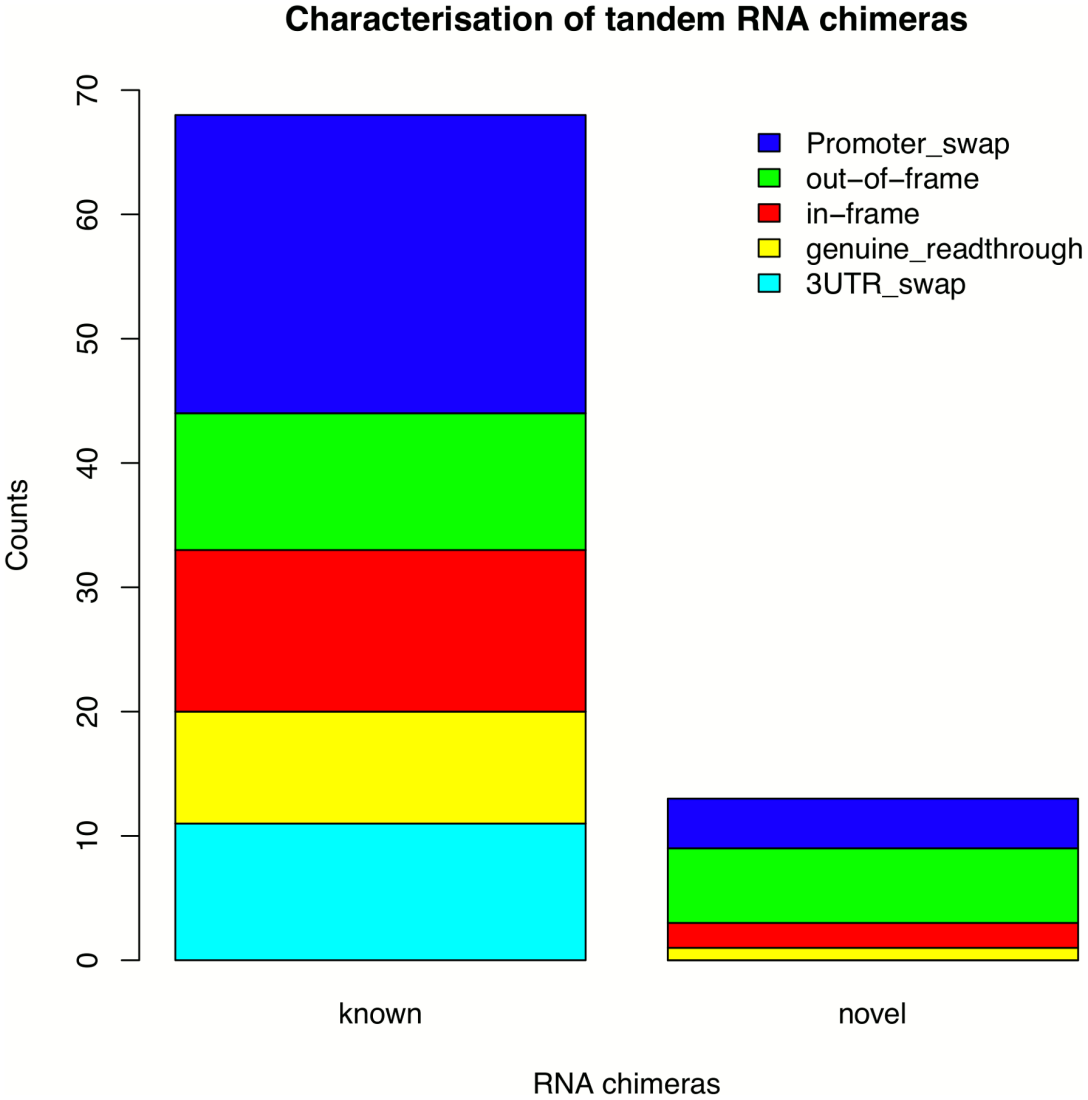
Characterization of tandem RNA chimeras. Barplot representing the frequency of the predicted structural classes for all tandem RNA chimeras. The most prevalent observed class is a promoter swap event.

We further classified the identified tandem RNA chimeras as “promoter-swap” events, “3′ UTR-swap” events and “genuine read-through” events. We considered a fusion as a promoter swap event if the coding sequence of the 3′ partner was placed under the promoter of the 5′ partner gene. Similarly, in 3′ UTR-swaps the coding sequence of the 5′ partner was placed under the control of the UTR of the 3′ partner. Fusions, where the whole coding sequences of both genes were conserved, were classified as “genuine read-through” events. Taking this approach, we identified 10 genuine read-through events, 28 promoter swap events, 11 3′ UTR swap events, 15 in-frame chimeras and 17 out-of-frame chimeras (Table S2 in File S1, Figure 1).

In addition to the tandem RNA chimeras we report 6 intrachromosomal fusions located on different strands, only one of which (*NAIP*->*OCLN*) was known [34] (Table S3 in File S1), and 15 interchromosomal fusions, 5 of which were previously described (Table S4 in File S1). While three of the chimeras (*ACTB*->*POTEM*, *ACTB->POTEE* and *SP100*->*HMGB1*) have been found in normal population [35], [36], three of the chimeras (*C2orf27A*->*NBEA*, *HILPDA*->*EFCAB3*, *FARSB*->*TRIM61*) were previously identified only in cancer samples [37]. This indicates that some chimeric RNAs present in normal samples can be considered as cancer specific due to the lack of appropriate controls.

**Figure 2:**
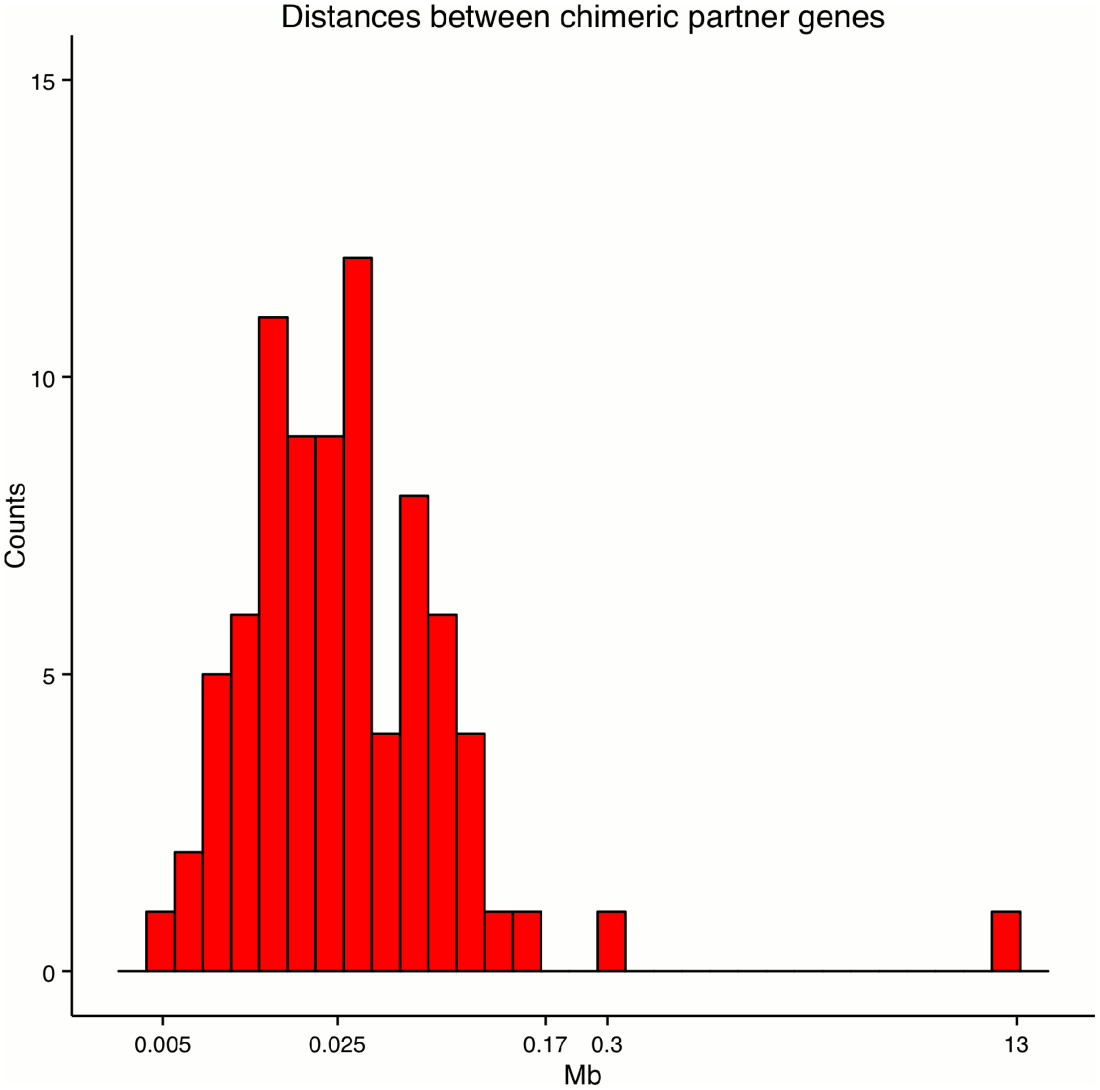
Distances between chimeric partner genes. Shown are distances between the tandem RNA chimeric partner genes in Mb. The majority of the distances are in the range between 5 kb and 170 kb.

We further calculated the distances between the breakpoints of the tandem RNA chimeras (Figure 2). The range varied between 5kb and 170 kb with a median distance of 25 kb except for some outliers: *COX5A*->*EDC3* with a distance of 0.3 Mb and *NRXN1*->*EIF2AK2* with a distance of 13 MB. As previously reported, tandem RNA chimeras tend to originate from genes residing closer on the genome [6]. In comparison to the tandem RNA chimeras for chimeric partner genes residing on different strands, we observed larger distances, which implies a different formation mechanism. The range of the distances between these genes was in the interval from 25 kb to 227 Mb with a median value of 2 MB.

### Frequency of chimeric transcripts in populations

**Figure 3:**
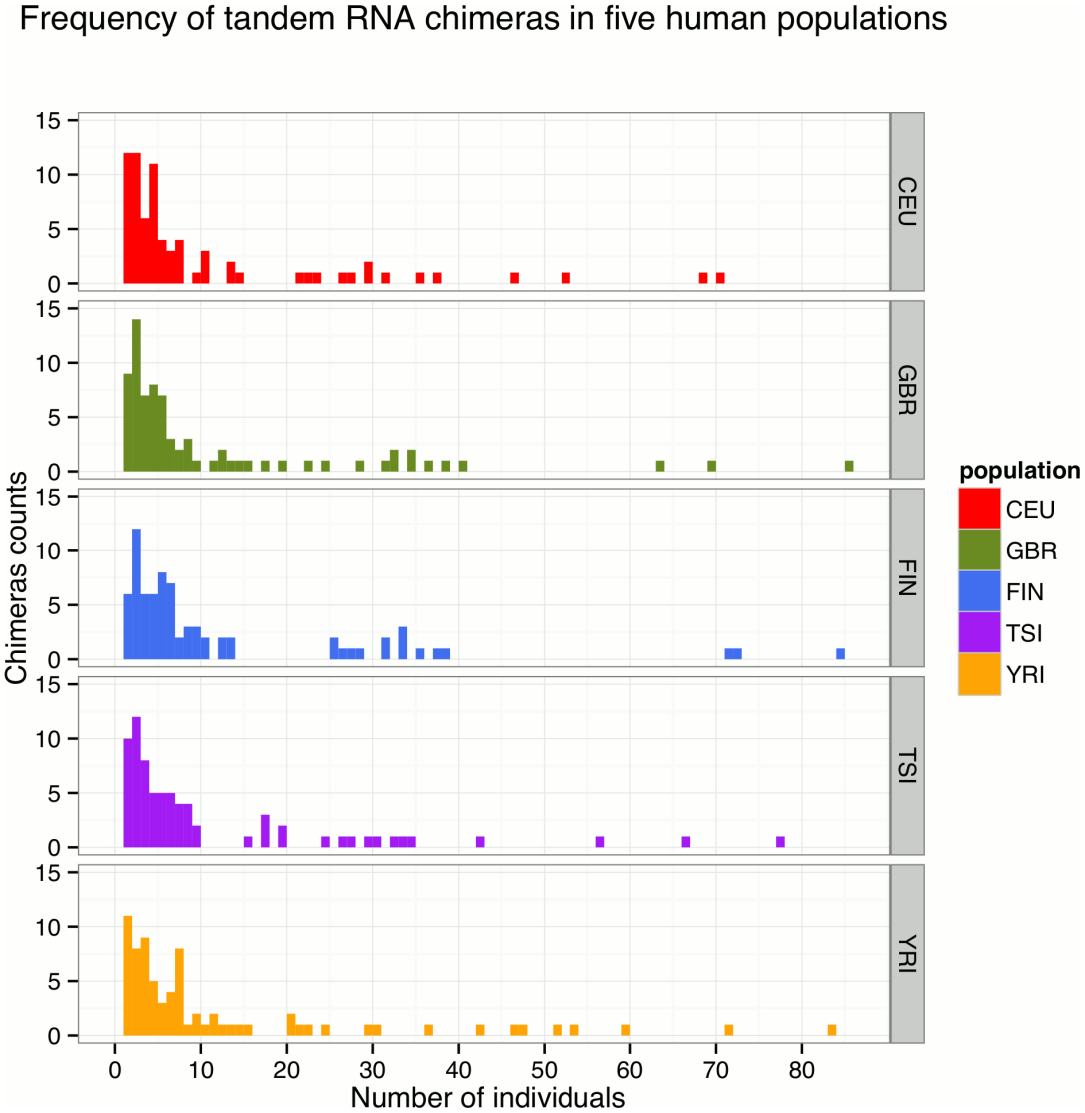
Tandem RNA chimeras frequency plots in five human populations. All populations show similar patterns with most of the chimeric transcripts found only in a few individuals.

**Figure 4:**
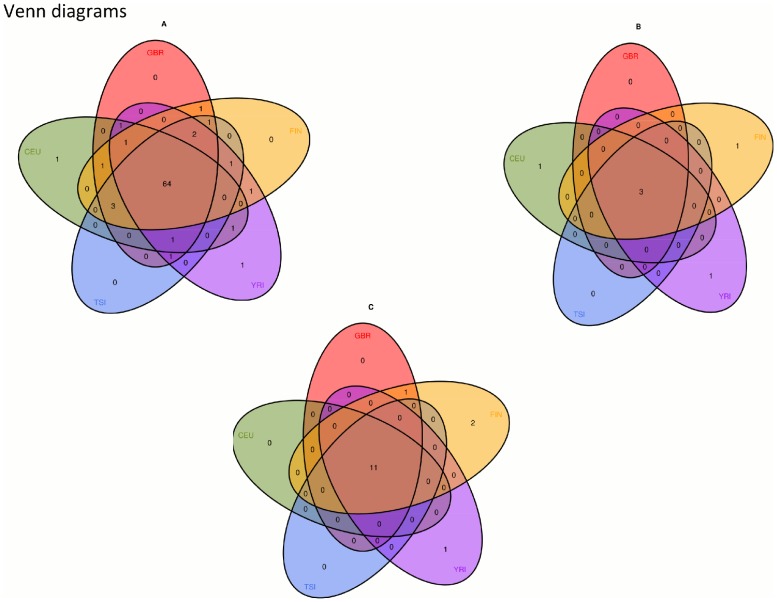
Venn diagrams. Venn diagrams displaying the relations between tandem RNA chimeras in five human populations (A); intrachromosomal chimeras located on different strands (B); and interchromosomal chimeras (C).

To assess the distribution of the RNA chimeras across populations, we calculated the frequency of the tandem RNA chimeras in the studied five populations. Frequency plots and venn diagram of the tandem RNA chimeras showed that most of the chimeric transcripts are found only in a few individuals (Figure 3 and Figure 4A). We further observed that while the high-frequency chimeric transcripts were present in all five studied populations, some rare chimeric RNAs were detected in single individuals as for example “*COX5A*->*EDC3*” and “*COPE*->*CERS1*” were discovered only in one individual of the YRI population and the CEU population respectively (Table S2 in File S1). Moreover, we detected similar expression values for the partner genes in the 5 human populations suggesting that the varying frequency of the chimeric transcripts in the populations is not due to expression differences (Table S5 in File S1). However, providing the small number of samples for each population we could not determine if these rare RNA chimeras are rather low frequent or population specific.

Similar trend in the population frequencies was revealed for the other two identified chimeric transcripts sets (intrachromosomal RNA chimeras located on different strands and interchromosmal RNA chimeras) as shown in the venn diagrams (Figure 4B–C).

### Exon exclusion pattern of tandem RNA chimeras

We further focused to study in more detail the tandem RNA chimeras as the most prevalent type amongst the identified RNA chimeras. In addition, the majority of the tandem RNA chimeras have been already confirmed in the literature. Moreover, tandem RNA chimeras are most likely to represent genuine read-through or intergenic splicing events rather than being a result from genomic structural variations. Furthermore, the data on structural variations available from the 1000 genome project [38] does not include inversions and translocations, therefore not allowing filtering of known structural variants in the intrachromosomal located on different strands and interchromosomal fusions data sets.

**Figure 5:**
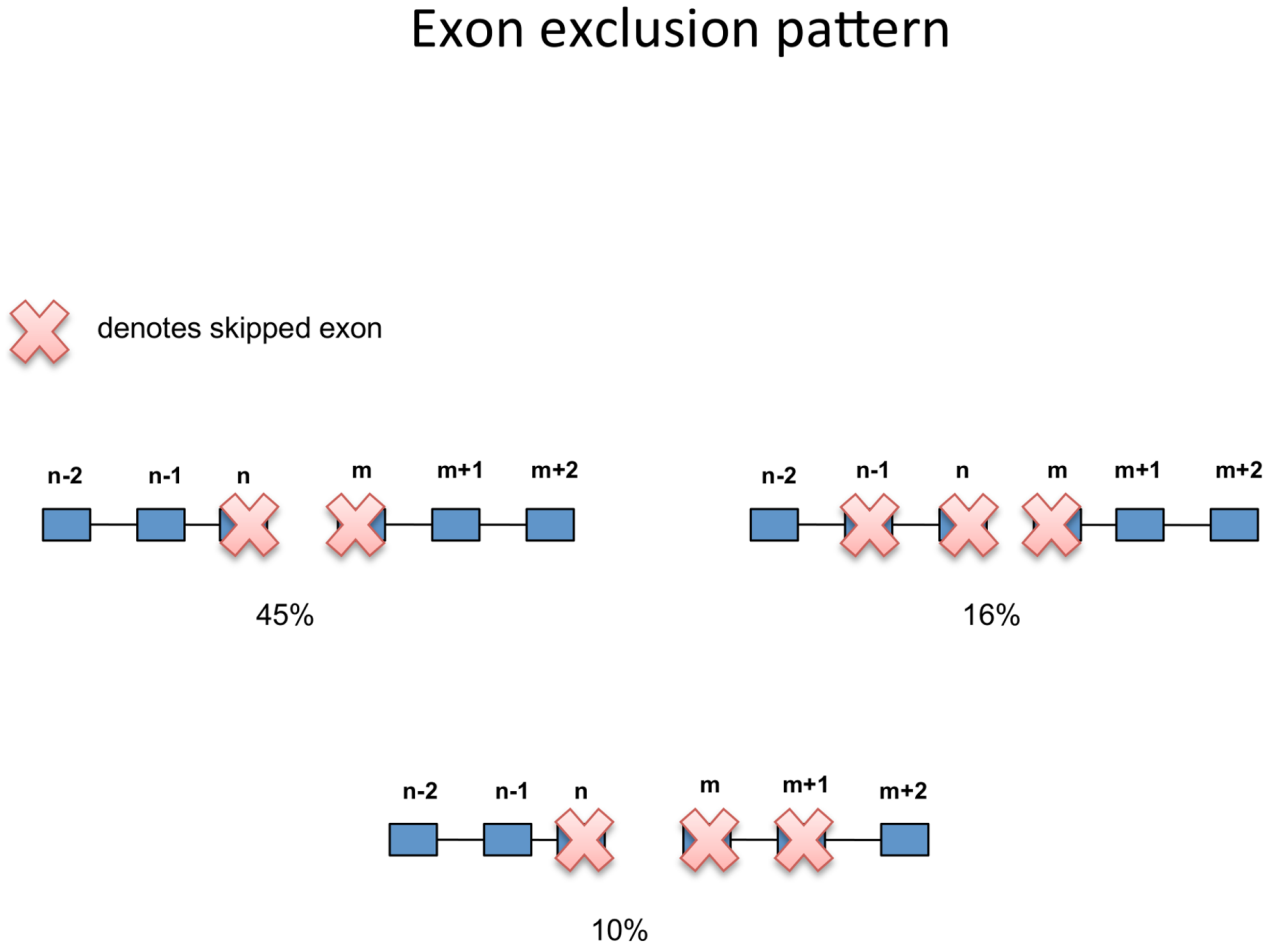
Exon exclusion pattern. Plot showing the proportions of the exon exclusion patterns in the tandem RNA chimeras. The numbers of the exons for the 5′ partner gene are counted from last to first, while the numbers of the exons for the 3′ partner gene are counted from first to last. The most abundant exon exclusion pattern involves the exclusion of the terminal exon of the 5′ partner gene and the first exon of the 3′ partner gene.

We further sought to study the exon exclusion pattern in the tandem RNA chimera formation. We took into account all possible fusion transcripts (shown in Table S6 in File S1). Then, we estimated the proportions of the excluded exons. Figure 5 shows that the most abundant exon exclusion pattern (45%) involves the exclusion of the terminal exon of the 5′ partner gene and the first exon of the 3′ partner gene, a pattern already observed previously [6], [7]. The next most common exon exclusion pattern (16%) represented an omission of the last two exons of the 5′ partner and the first exon of 3′ partner, followed by the exclusion of the last terminal exon of the 5′ partner gene and the first two exons of the 3′ partner gene in 10% of the cases. Therefore, the last two exons of the 5′ partner gene and the first two exons of the 3′ partner genes are most likely to be spliced out during the tandem RNA chimera formation.

### Tandem RNA chimeras are lowly expressed genes

**Figure 6:**
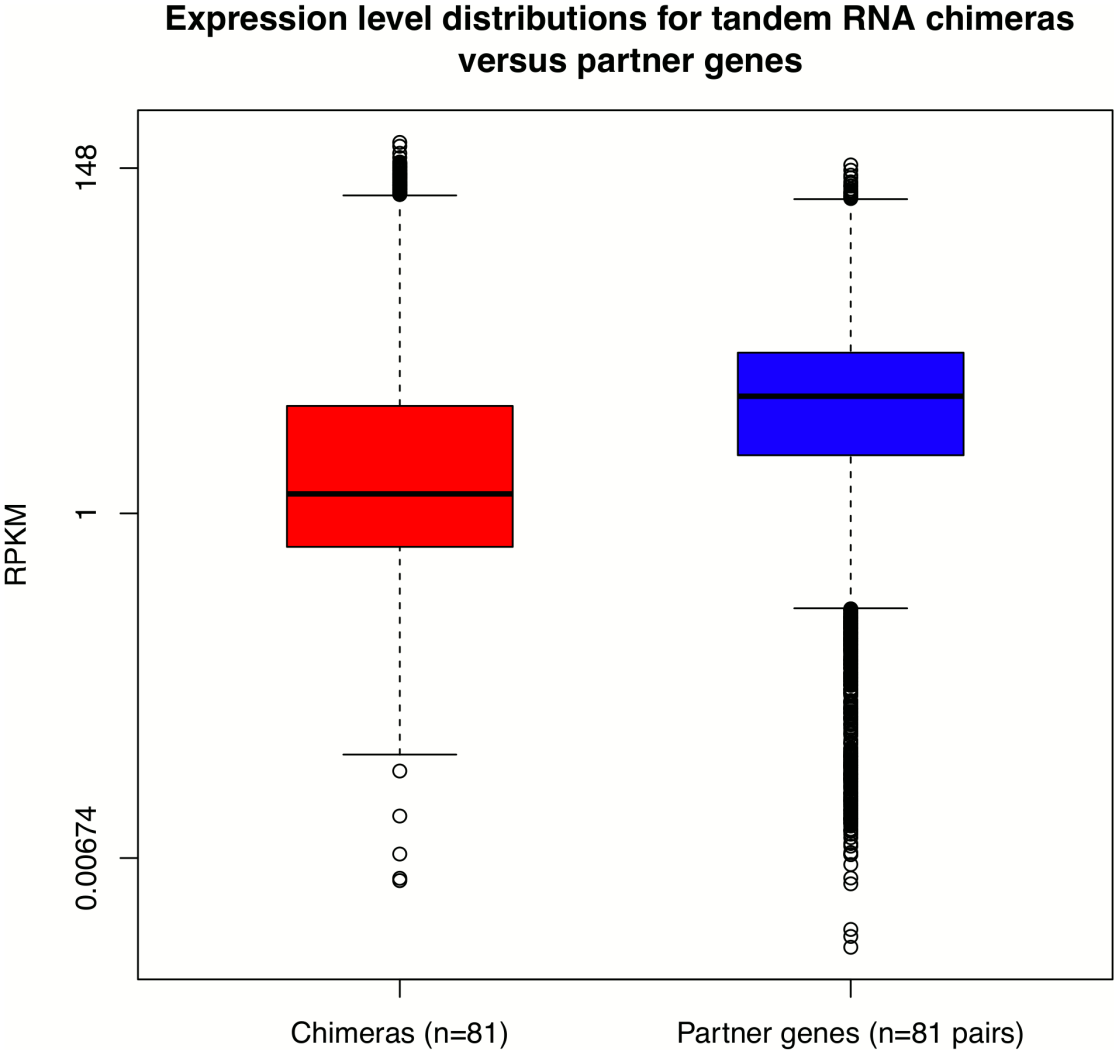
Expression level distributions of tandem RNA chimeras and their partner genes. RNA chimeras are lower expressed than their partner genes.

Next, we explored in more detail the gene expression level of the tandem RNA chimeras. We created a custom gtf file with added all possible transcription combinations of the identified tandem RNA chimeras. Then, we compared the distribution of the summarized per gene expression values of the tandem RNA chimeras to the distribution of the expression values of the partner genes. Furthermore, given the fact that the chimeric transcripts were identified only in subset of individuals from each population, we compared only the expression values estimated in these individuals. As it shown in Figure 6, we observed that the tandem RNA chimeras are at lower abundances compared to the transcripts of the partner genes (Kolmogorov-Smirnov test, D = 0.3262, p-value<2.2e-16) with median values of 1.33 and 5.45 for the tandem RNA chimeras and for the partner genes respectively. This result is in agreement with a previous report, where the expression values of chimeric genes were estimated by a different approach using the reads located at the fusion junction [39]. However, it is worth mentioning that Frenkel-Morgenstern estimations were performed on datasets of chimeric transcripts, which included cancer specific fusions.

### Long introns are overrepresented in tandem RNA chimeras

**Figure 7:**
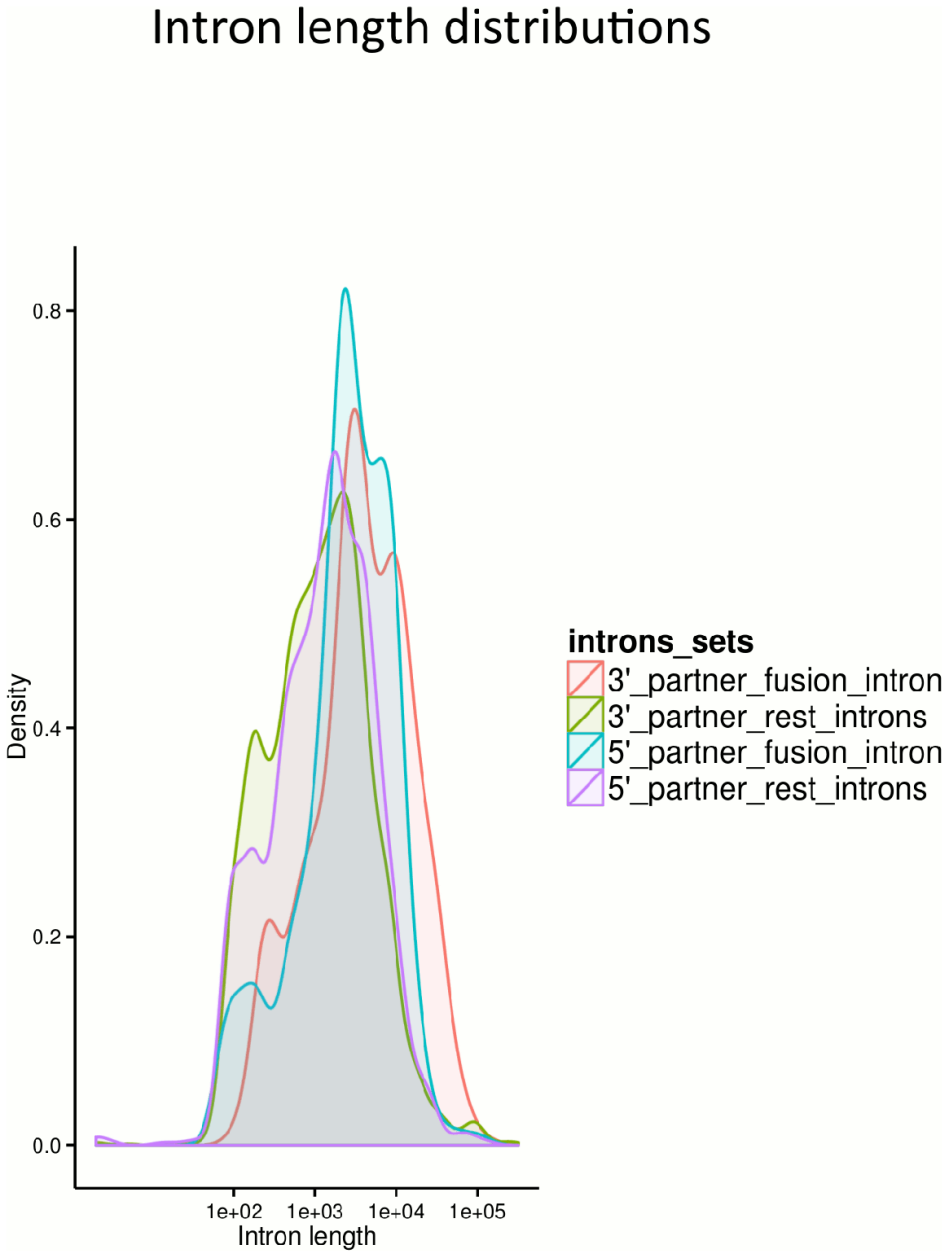
Intron length density plots. Density plots showing the distributions of the fusion 5′ and 3′ introns length versus the length of the rest of the introns comprising the fusion partner genes. Observed is a notable higher proportion of larger introns at the breakpoint.

To check the possibility if the size of the exons and introns in the proximity of the fusion breakpoint could influence the RNA chimera formation, we compared the distributions of the exons and introns lengths located at the fusion boundaries to the lengths of the rest of the introns and exons comprising the fusion partner genes. While we did not observe a notable difference between the exon lengths (data not shown), we found that the chimeric genes tend to have larger introns at the fusion breakpoint as compared to the rest of the introns comprising the partner genes, with estimated Kolmogorov-Smirnov tests, D = 0.278, p-value<2.2e-16 and D = 0.389, p-value<2.2e-16 for the 5′ intron and 3′ intron respectively (Figure 7). We observed median lengths of 2516 nt, 3734 nt for the intron following the fusion exon of the 5′ partner gene and for the intron proceeding the fusion exon of the 3′ partner gene respectively, as compared to median lengths of 1400 and 1164 estimated for the rest of the introns of the 5′ and 3′ partner genes.

### Association of tandem RNA chimeras formation with genetic variants

To explore further the mechanism of tandem RNA chimera formation, we took advantage of the available genomic data from the same individuals provided by the 1000 genome project, with functional re-annotation with Gencode v12 as described in [16].

To check the hypothesis that deletions or mutation in the poly(A) signal of the 5′ partner gene drive the tandem RNA chimera formation, we inspected the poly(A) signals and sites as described in Methods. We did not observed in any 5′ partner genes deletions in the poly(A) signals. Two genes (LRRC33 and SDHD) out of 81 5′ partner genes were detected to have single nucleotide polymorphisms (A->G) in their poly(A) signal (rs77865386, rs17113461 respectively). Similarly, we could not identify any genes bearing deletions in their poly(A) sites. Only seven genes were identified to have single nucleotide polymorphisms in these sites (Table S7 in File S1).

Since the identified genetic variants in poly A signal or sites could not explain the mechanism of chimeras formation for the majority of the identified tandem RNA chimeras, we took an approach similar to genome wide association studies, where for each chimeric RNA, the cases consisted of individuals with chimera and controls were represented by individuals without chimera. For our analysis, we selected only fusions with frequency higher than 5% in all populations (37 chimeras) and SNPs with MAF >5%.

By using this approach we identified 54 variants associated with the formation of 3 chimeras (Table S8 in File S1). All of the variants were located in intronic regions and more specifically, in introns surrounding the exon involved in the fusion. For all variants located at PABPC1 (Poly(A)denylate-binding protein 1) binding sites, which potentially could represent poly(A) sites or signals, we compared these to annotated from Gencode v12 or predicted poly(A) sites and signals found in intronic genomic locations. We did not observe any overlap. We further inspected the identified variants by regional association plots (Figure S1 in File S1, Figure S2 in File S1 and Figure S3 in File S1) and subsequently selected for each chimera the most significant variants having the lowest p-value (Table 1). Two out of the three variants rs9945924 and rs4148876, have been already reported to play an important role in controlling alternative splicing and disease progression [40].

**Table 1:** The most significant SNPs associated with RNA chimeras formation.

Gene	Chr	Position	SNP	p_value	adjusted p-value	Ref/Alt	Odd ratio	95% CI
GNG5	1	84965623	rs56212819	1.37E-04	2.87E-03	C/T	2.01	1.33–3.04
HMSD	18	61620766	rs9945924	5.55E-17	8.66E-15	G/A	4.79	3.22–7.14
TAP2	6	32796793	rs4148876	2.20E-16	4.77E-14	G/A	18.75	8.21–42.82

## Discussion

Chimeric transcripts, which contribute to the complexity of the human transcriptome, have already drowned the attention of the scientific community. However, the efforts to study this phenomenon are hampered by the lack of large datasets of normal populations.

Here, by combining expression and genetic variation data from the same individuals in a large cohort of samples, we identified and characterized chimeric transcripts in normal population. We focused on the most prevalent class of chimeric transcripts - tandem RNA chimeras. As a proof of concept most of the tandem RNA chimeras we identified were already present in public databases and in the literature, however we found 13 novel tandem RNA chimeras, demonstrating that there are more still to be discovered in normal populations. Our results showed prevalence of promoter swap events. An opposite trend was reported in cancer samples, where the most prevalent class of fusion transcripts occurs within the 3′ UTR sequence [41].

We observed varying frequency of RNA chimeras between individuals, which confirms that chimeric transcripts represent another layer of transcriptional variation existing in the human population.

Our results are in agreement with other groups [6], [7] showing evidence that the tandem RNA chimeras originate from genes that tend to reside closer on the genome. Perturbations in co-transcriptional coupling of terminal intron splicing and 3′ end processing [42]–[44] could explain the observations found in this and previous studies [6], [7], with the most frequent pattern featuring splicing out the terminal exon of the 5′ partner and the first exon of the 3′ partner. Furthermore, our results demonstrated prevalence of long introns in the vicinity of the 5′ and 3′ fusion exons. A long intron at the fusion breakpoint could allow enough time for the intergenic splicing to compete with the cis-splicing machinery for the 5′ unsaturated splice donor site. It has been known that long introns can promote alternative splicing [45]. Moreover, a study on human cells expressing modified *Sp1* transgenes showed evidence that pausing of RNA polymerase II and presence of long intron promotes trans-splicing of genes residing on different chromosomes [46]. Herein, we confirmed this observation for tandem RNA chimera formation by using large scale high-throughput data.

Contrary to some reports [14], which suggests that deletions at poly(A) signals or poly(A) sites are responsible for the tandem RNA chimera formation, we could not identify such events. We observed genetic variants in poly A sites and signals in 9 of the 81 5′ partner genes, however these changes are unlikely to account for the mechanism behind the tandem RNA chimera origin. We do not exclude the possibility that other 3′ termination signal or trans-factors could play a role in the chimera formation.

By performing genetic association studies we were able to identify three variants associated with the presence of tandem RNA chimeras. All variants were located in intronic regions. Introns have increasing attention from the scientific community for their role in pre-mRNA splicing [47]. Reassuringly, two of the variants were already reported to play an important role in alternative splicing [48]. Thus, our results show that variants affecting splicing contribute to the RNA chimera origin.

By utilizing RNA binding sites and regulatory features genomic regions data we observed that two of the three variants are located in PABPC1 binding sites. PABPC1 has been mostly known to bind poly(A) tail and is involved in NMD pathway [49]. Several groups report involvement of PABPC1 in protein complexes playing a role in mRNA stability [50], [51]. Thus, PABPC1 could be also involved in RNA metabolism including splicing. More studies are needed to identify cis- and trans-factors involved in the formation of tandem RNA chimeras.

Our observations also demonstrate that many chimeras naturally occurring in normal populations have been mistaken for cancer-specific. For example *NRXN1->EIF2AK2*, *PRIM1*->*NACA* [52], *PRKAA1*->*TTC33*, *CTBS*->*GNG5* [53], *SLC35A3*->*HIAT1* [37] and *UBE2J2*->*FAM132A* [54]. In addition, the chimeras implying inversions were also considered as cancer-specific or being related to other diseases. For example *PPIP5K1*->*CATSPER2* and *YARS2*->*NAP1L1* [54] have been predicted in melanoma samples. The chimeras *TRIP12*->*SLC16A14* and *TFG*->*GPR128* have been found in cancer patients with clear cell renal carcinoma [55]. Nevertheless, another group reported identifying *TFG*->*GPR128* in healthy individuals [56]. Two of the chimeras implying inversion *MAPKAPK5*->*ACAD10* and *POLR1A*->*REEP1* have been detected in autistic individuals [57]. These findings suggest that lack of rigorous normal controls could hamper the identification of genuine cancer-specific fusions. In addition, we noticed that 4% of the fusion genes identified in a clear cell renal cell carcinoma dataset represented RNA chimeras found in this study (data not shown). On the other hand we have to take into account that perturbations in the RNA chimera formation naturally occurring in normal population may lead to cancer progression. Such notion is supported by recent reports on differential expression in tandem RNA chimeras in tumor versus normal samples [58] or on identified tandem chimeric RNAs in cancer cells without an evidence for genomic rearrangements [59]. This complexity adds another challenge to the cancer biomarker discovery. In conclusion, more studies are needed to understand the mechanisms behind the chimeric transcripts regulation in healthy individuals and the perturbations of these naturally occurring events, which could lead to cancer.

## Supporting Information

File S1 **File includes Figures S1–S3 and Tables S1–S8.** Figure S1: Regional association gene plots for CTBS->GNG5 chimera. Results are shown in the region flanking 100 kb both sides of the index SNPs. The marker SNPs are shown in purple and the color of the dots represent the degree of linkage disequilibrium (based on r2) in relation to the index SNP based on the March 2012 release of the 1000 Genomes data in European population. Figure S2: Regional association gene plots for HMSD->SERPINB8 chimera. Results are shown in the region flanking 100 kb both sides of the index SNPs. The marker SNPs are shown in purple and the color of the dots represent the degree of linkage disequilibrium (based on r2) in relation to the index SNP based on the March 2012 release of the 1000 Genomes data in European population. Figure S3: Regional association gene plots for TAP2->HLA-DOB chimera. Results are shown in the region flanking 100 kb both sides of the index SNPs. The marker SNPs are shown in purple and the color of the dots represent the degree of linkage disequilibrium (based on r2) in relation to the index SNP based on the March 2012 release of the 1000 Genomes data in European population. Table S1: Intrachromosomal RNA chimeras residing on the same strand, which orientation implies inversion. Table S2: Identified tandem RNA chimeras located on the same strand. Table S3: Identified intrachromosomal RNA chimeras located on different strands. Table S4: Identified interchromosomal RNA chimeras. Table S5: Gene expression values estimated for tandem RNA chimeras partner genes in each human population. Table S6: All annotated transcripts and exons residing at the fusion junction for the identified tandem RNA chimeras. Table S7: Genetic variants found in poly(A) sites of tandem RNA chimeras upstream genes. Table S8: All identified genetic variants associated with tandem RNA chimera formation.
(PDF)
