# Supplementary material for: Tandem RNA Chimeras Contribute to Transcriptome Diversity in Human Population and Are Associated with Intronic Genetic Variants
Source: PLoS One. 2014 Aug 18;9(8):e104567. doi: 10.1371/journal.pone.0104567 (PMC4136775; doi:10.1371/journal.pone.0104567)
Supplement: File S1 — File includes Figures S1–S3 and Tables S1–S8. Figure S1: Regional association gene plots for CTBS->GNG5 chimera. Results are shown in the region flanking 100 kb both sides of the index SNPs. The marker SNPs are shown in purple and the color of the dots represent the degree of linkage disequilibrium (based on r2) in relation to the index SNP based on the March 2012 release of the 1000 Genomes data in European population. Figure S2: Regional association gene plots for HMSD->SERPINB8 chimera. Results are shown in the region flanking 100 kb both sides of the index SNPs. The marker SNPs are shown in purple and the color of the dots represent the degree of linkage disequilibrium (based on r2) in relation to the index SNP based on the March 2012 release of the 1000 Genomes data in European population. Figure S3: Regional association gene plots for TAP2->HLA-DOB chimera. Results are shown in the region flanking 100 kb both sides of the index SNPs. The marker SNPs are shown in purple and the color of the dots represent the degree of linkage disequilibrium (based on r2) in relation to the index SNP based on the March 2012 release of the 1000 Genomes data in European population. Table S1: Intrachromosomal RNA chimeras residing on the same strand, which orientation implies inversion. Table S2: Identified tandem RNA chimeras located on the same strand. Table S3: Identified intrachromosomal RNA chimeras located on different strands. Table S4: Identified interchromosomal RNA chimeras. Table S5: Gene expression values estimated for tandem RNA chimeras partner genes in each human population. Table S6: All annotated transcripts and exons residing at the fusion junction for the identified tandem RNA chimeras. Table S7: Genetic variants found in poly(A) sites of tandem RNA chimeras upstream genes. Table S8: All identified genetic variants associated with tandem RNA chimera formation. (PDF) [file pone.0104567.s001.pdf]

Table S1

Chimera	Class	Chr	Strand	Bp gene1	Bp gene 2	Novelty	CEU	GBR	FIN	TSI	YRI
**MAPKAPK5->ACAD10**	promoter_swap	chr12	+	112308984	112182447	Holt et al, 2012	0	1	0	0	0
**POLR1A->REEP1**	promoter_swap	chr2	-	86292397	86509365	Holt et al, 2012	0	1	0	0	0
**PPIP5K1->CATSPER2**	in-frame	chr15	-	43857065	43924561	Kinsella et al, 2011	18	16	14	17	8
**TFG->GPR128**	in-frame	chr3	+	100438902	100348442	Chase et al, 2010	0	2	0	2	0
**TRIP12->SLC16A14**	in-frame	chr2	-	230723488	230902247	Hakimi et al, 2013	0	1	0	0	0
**TYK2->CDC37**	in-frame	chr19	-	10488890	10504113	novel	0	0	0	0	1
**VBP1->BRCC3**	in-frame	chrX	+	154444852	154300602	novel	0	0	0	0	1
**YARS2->NAP1L1**	out-of-frame	chr12	-	32900010	76446890	Kinsella et al, 2011	3	3	1	1	3

Table S2

Chimera	Class	Chr	Strand	Bp gene1	Bp gene 2	Novelty	CEU	GBR	FIN	TSI	YRI
**AP5S1->MAVS**	promoter_swap	chr20	+	3802940	3835205	AceView	1	2	2	2	3
**ARHGAP19->SLIT1**	out-of-frame	chr10	-	98988904	98924647	AceView	1	1	1	0	1
**C11orf48->INTS5**	in-frame	chr11	-	62430708	62417471	AceView	3	0	0	0	2
**C7orf55->LUC7L2**	in-frame	chr7	+	139030367	139060808	AceView	5	4	3	2	4
**CCL22->CX3CL1**	out-of-frame	chr16	+	57394472	57413546	novel	3	1	2	2	0
**CENPE->BDH2**	promoter_swap	chr4	-	104029960	104017431	novel	2	3	3	1	2
**CHURC1->FNTB**	out-of-frame	chr14	+	65390844	65470967	AceView	1	7	6	7	8
**CLN6->CALML4**	out-of-frame	chr15	-	68521840	68489966	AceView	29	19	31	29	24
**CNPY2->CS**	promoter_swap	chr12	-	56708635	56680429	AceView	5	2	3	3	7
**COPE->CERS1**	out-of-frame	chr19	-	19010682	18995076	AceView	1	0	0	0	0
**CORO7->PAM16**	3UTR_swap	chr16	-	4405287	4393292	AceView	6	6	7	3	2
**COX5A->EDC3**	in-frame	chr15	-	75230256	74932960	novel	0	0	0	0	1
**CTBS->GNG5**	3UTR_swap	chr1	-	85028940	84967653	AceView	31	24	33	33	29
**CTSC->RAB38**	in-frame	chr11	-	88033698	87883123	Nacu et al, 2011	2	2	4	2	1
**DDX5->POLG2**	in-frame	chr17	-	62496667	62489138	AceView	2	1	5	4	4
**ELAVL1->TIMM44**	out-of-frame	chr19	-	8038609	8006082	novel	0	1	1	0	0
**ENTPD1->C10orf131**	promoter_swap	chr10	+	97624618	97671058	novel	4	6	9	6	6
**FAM18B2->CDRT4**	genuine read-through	chr17	-	15449099	15343599	AceView	9	28	33	19	21
**FKBP1A->SDCBP2**	promoter_swap	chr20	-	1373478	1301079	AceView	7	14	25	15	15
**GPI->PDCD2L**	in-frame	chr19	+	34887562	34895554	AceView	26	34	27	32	30
**HACL1->COLQ**	3UTR_swap	chr3	-	15604865	15531144	AceView	2	2	6	3	7
**HAUS4->PRMT5**	genuine read-through	chr14	-	23416380	23397824	AceView	2	2	5	3	2
**HMSD->SERPINB8**	promoter_swap	chr18	+	61627509	61645533	AceView	29	32	35	34	59
**HSPE1->MOBKL3**	promoter_swap	chr2	+	198367852	198388348	AceView	4	9	6	7	13
**IFNAR2->IL10RB**	3UTR_swap	chr21	+	34625135	34640699	AceView	2	2	4	2	3
**IFRD1->C7orf53**	promoter_swap	chr7	+	112112916	112124867	AceView	1	3	2	0	0
**ISY1->RAB43**	3UTR_swap	chr3	-	128853675	128814012	AceView	5	1	4	1	1
**JAK3->INSL3**	out-of-frame	chr19	-	17940917	17927868	novel	0	4	1	5	5
**KIAA0101->CSNK1G1**	genuine read-through	chr15	-	64668942	64592922	AceView	0	0	2	2	7
**KIAA0494->ATPAF1**	out-of-frame	chr1	-	47148972	47131036	AceView	1	3	5	17	7
**LMAN2->MXD3**	in-frame	chr5	-	176761285	176738485	Nacu et al, 2011	6	1	4	1	1
**LRRC33->PIGX**	promoter_swap	chr3	+	196381518	196443729	AceView	2	3	5	4	3
**LSP1->TNNT3**	genuine read-through	chr11	+	1908806	1944087	novel	4	7	5	6	9
**MED8->ELOVL1**	promoter_swap	chr1	-	43850717	43831294	AceView	10	12	12	3	6
**METTL10->FAM53B**	promoter_swap	chr10	-	126448886	126395455	AceView	2	2	2	2	3
**METTL21B->TSFM**	promoter_swap	chr12	+	58166911	58176893	AceView	3	8	9	5	10
**NDUFA13->YJEFN3**	in-frame	chr19	+	19638579	19645843	AceView	10	11	6	8	3
**NDUFB8->SEC31B**	genuine read-through	chr10	-	102286156	102276717	AceView	35	38	38	30	46
**NRXN1->EIF2AK2**	promoter_swap	chr2	-	50574005	37374964	Francis et al, 2012	37	34	33	26	53
**NSUN4->FAAH**	in-frame	chr1	+	46818700	46867763	AceView	3	4	10	7	2
**PEX26->TUBA8**	promoter_swap	chr22	+	18568024	18604246	AceView	0	1	0	2	2
**PFKFB4->SHISA5**	genuine read-through	chr3	-	48559419	48538726	AceView	1	2	2	2	0
**PKHD1L1->EBAG9**	promoter_swap	chr8	+	110535615	110563039	novel	4	2	2	4	1
**PLEKHO2->ANKDD1A**	promoter_swap	chr15	+	65153774	65214120	AceView	4	4	6	1	0
**POLA2->CDC42EP2**	promoter_swap	chr11	+	65063461	65088015	AceView	4	4	10	6	11
**PPRC1->NOLC1**	out-of-frame	chr10	+	103909086	103916776	novel	2	5	3	2	3
**PRH1->PRR4**	3UTR_swap	chr12	-	11034816	11001006	AceView	14	13	6	17	12
**PRIM1->NACA**	3UTR_swap	chr12	-	57127931	57108471	Francis et al, 2012	21	31	31	19	20
**PRKAA1->TTC33**	promoter_swap	chr5	-	40764616	40747121	Plebani et al., 2012	1	4	2	1	1
**PRR11->C17orf71**	out-of-frame	chr17	+	57275150	57289702	AceView	3	4	4	2	4
**PRR13->PCBP2**	genuine read-through	chr12	+	53837557	53848510	AceView	4	5	9	5	20
**PXMP2->PGAM5**	promoter_swap	chr12	+	133266962	133291444	AceView	5	3	5	3	7
**RBM14->RBM4**	promoter_swap	chr11	+	66384528	66407171	AceView	68	63	71	56	71
**RNASET2->RPS6KA2**	3UTR_swap	chr6	-	167352383	167271747	AceView	22	32	28	27	36
**RRM2->C2orf48**	promoter_swap	chr2	+	10269281	10281981	AceView	70	85	84	77	83
**S1PR2->DNMT1**	out-of-frame	chr19	-	10341880	10292753	AceView	4	5	1	3	6
**SDHAF2->C11orf66**	3UTR_swap	chr11	+	61205585	61252160	AceView	0	5	7	1	3
**SDHD->TEX12**	out-of-frame	chr11	+	111959735	112041142	AceView	7	5	6	5	5
**SLC35A3->HIAT1**	out-of-frame	chr1	+	100483371	100515465	Salzman et al, 2012	4	2	3	5	3
**SLC39A1->CRTC2**	in-frame	chr1	-	153934696	153927642	AceView	23	22	26	17	42
**SLC43A3->PRG2**	genuine read-through	chr11	-	57176648	57157429	AceView	52	69	72	66	51
**SMG1->ARL6IP1**	promoter_swap	chr16	-	18823083	18810156	novel	0	0	2	0	1
**SNTB2->VPS4A**	3UTR_swap	chr16	+	69333677	69349911	AceView	7	5	2	4	9

**SUMO2->HN1**	promoter_swap	chr17	-	73170847	73144766	Prakash et al, 2010	1	2	4	1	1
**SYNJ2BP->COX16**	in-frame	chr14	-	70842393	70809446	AceView	2	1	2	1	2
**TAGLN2->CCDC19**	promoter_swap	chr1	-	159895240	159863095	Nacu et al, 2011	10	15	12	7	5
**TAP2->HLA-DOB**	out-of-frame	chr6	-	32797177	32783090	AceView	4	8	8	8	1
**TMBIM4->LLPH**	genuine read-through	chr12	-	66531740	66522892	AceView	1	2	0	0	3
**TNFAIP8L2->SCNM1**	promoter_swap	chr1	+	151129198	151138947	AceView	46	40	37	42	47
**TOMM5->FBXO10**	genuine read-through	chr9	-	37588808	37541771	AceView	1	1	3	8	4
**TOPORS->DDX58**	promoter_swap	chr9	-	32550772	32500937	AceView	4	2	0	2	2
**TPD52L2->DNAJC5**	promoter_swap	chr20	+	62505169	62559688	AceView	2	5	5	1	4
**TSC22D4->C7orf61**	in-frame	chr7	-	100074900	100061309	AceView	6	8	13	6	6
**TSTD1->F11R**	in-frame	chr1	-	161008670	160971143	AceView	27	36	25	24	22
**UBA2->WTIP**	in-frame	chr19	+	34957919	34981281	novel	13	6	8	8	7
**UBE2J1->GABRR2**	out-of-frame	chr6	-	90039502	90009584	novel	1	4	2	3	7
**UBE2J2->FAM132A**	out-of-frame	chr1	-	1192372	1179877	Kinsella et al, 2012	0	3	1	1	0
**UCHL3->LMO7**	out-of-frame	chr13	+	76169126	76195899	novel	13	17	13	9	14
**UQCRQ->LEAP2**	3UTR_swap	chr5	+	132203372	132209642	Kinsella et al, 2012	2	3	5	6	7
**VKORC1->PRSS53**	in-frame	chr16	-	31104633	31099147	AceView	3	2	1	4	1
**ZNF343->SNRPB**	promoter_swap	chr20	-	2473345	2448404	AceView	7	12	8	9	11

Table S3

Chimera	Class	Chr	Bp gene 1	Strand gene1	Bp gene 2	Strand gene2	Novelty	CEU	GBR	FIN	TSI	YRI
**C6orf72->PPIL4**	3UTR_swap	chr6	149887670	+	149862567	-	novel	0	0	1	0	0
**NAIP->OCLN**	Promoter_swap	chr5	70275696	-	68830521	+	Courseaux et al., 2003	15	23	13	10	30
**SMC4->BCL6**	Promoter_swap	chr3	160117537	+	187452695	-	novel	0	0	0	0	1
**ZNF175->CTU1**	in-frame	chr19	52085135	+	51602396	-	novel	1	0	0	0	0
**EDARADD->ENO1**	3UTR_swap	chr1	236646645	+	8928116	-	novel	18	18	11	17	16
**BPTF->LRRC37A3**	Promoter_swap	chr17	65822452	+	62894480	-	novel	9	13	7	10	9

Table S4

Fusion	Class	Chr gene1	Bp gene1	Strand gene 1	Chr gene2	Bp gene 2	Strand gene 2	Novelty	CEU	GBR	FIN	TSI	YRI
**ACTB->POTEM**	3UTR_swap	chr7	5568295	-	chr14	19988515	-	Lee et al., 2006	16	15	18	19	13
**ACTB->POTEE**	in-frame	chr7	5568201	-	chr2	5568178	+	Lee et al., 2006	19	22	23	19	27
**AIG1->PARL**	in-frame	chr6	143654582	+	chr3	183584546	-	novel	5	4	5	6	7
**ARL4A->MTHFD1L**	out-of-frame	chr7	12728059	+	chr6	151257940	+	novel	0	1	1	0	0
**C1orf189->TOX**	in-frame	chr1	154172935	-	chr8	59872567	-	novel	0	0	0	0	1
**C2orf27A->NBEA**	Promoter_swap	chr2	132508207	+	chr13	35630149	+	Salzman et a.l, 2011	9	3	7	4	5
**HILPDA->EFCAB3**	out-of-frame	chr7	128096077	+	chr17	60491093	+	Kinsella et al., 2011	24	17	25	19	18
**EEF1A1->XPOT**	out-of-frame	cjhr6	74229188	-	chr12	64813850	+	novel	0	0	1	0	0
**FARSB->TRIM61**	in-frame	chr2	223513459	-	chr4	165890827	-	Edgren et al., 2011	13	18	19	21	30
**NHP2L1->LLPH**	Promoter_swap	chr22	42076248	-	chr12	66522893	-	novel	3	4	3	5	17
**PRKCB->YBX1**	in-frame	chr16	24185897	+	chr1	43162380	+	novel	0	0	1	0	0
**RHOQ->LRR1**	in-frame	chr2	46808306	+	hr14	50069088	+	novel	3	4	3	6	11
**SAV1->GYPE**	in-frame	chr14	51131897	-	chr4	144801662	-	novel	38	62	43	46	46
**SP100->HMGB1**	in-frame	chr2	231379941	+	chr13	31037489	-	Guldner et al., 1999	8	13	9	11	12
**ZNF562->RBAK**	3UTR_swap	chr19	9760594	-	chr7	5103326	+	novel	1	3	4	3	2

Table S5

Chimera	Gene	Gene_ID	Mean_RPKM_GBR	Mean_RPKM_YRI	Mean_RPKM_TSI	Mean_RPKM_CEU	Mean_RPKM_FIN
**AP5S1->MAVS**	AP5S1	ENSG00000125843.6	5.906761468	5.996625809	5.888750269	5.987959396	5.611472032
**AP5S1->MAVS**	MAVS	ENSG00000088888.12	7.505470596	10.01010097	7.972410151	6.677136989	8.187470011
**ARHGAP19->SLIT1**	SLIT1	ENSG00000187122.9	0.709072021	0.764946461	0.635082763	0.881844692	0.836355653
**ARHGAP19->SLIT1**	ARHGAP19	ENSG00000213390.4	13.01433798	16.89458074	12.96258149	11.6073778	12.10698701
**C11orf48->INTS5**	INTS5	ENSG00000185085.2	5.853537681	5.805672674	6.284345226	5.889425604	5.994211895
**C11orf48->INTS5**	C11orf48	ENSG00000162194.8	70.71776774	66.73664255	73.62568718	68.67449168	68.19640529
**C7orf55->LUC7L2**	C7orf55	ENSG00000164898.8	5.676156521	5.410928876	5.844953763	6.470463088	5.396266221
**C7orf55->LUC7L2**	LUC7L2	ENSG00000146963.10	30.25117557	28.91158122	28.33275323	27.34069573	27.73785207
**CCL22->CX3CL1**	CCL22	ENSG00000102962.4	268.799309	249.8187332	288.0398444	372.219261	486.9927294
**CCL22->CX3CL1**	CX3CL1	ENSG00000006210.6	0.729051011	0.301538584	0.323854387	0.790080198	0.683067284
**CENPE->BDH2**	BDH2	ENSG00000164039.10	9.337327894	7.997691629	8.275214763	8.401546934	9.363227695
**CENPE->BDH2**	CENPE	ENSG00000138778.6	23.30811509	18.85819425	23.07119372	22.23271485	24.52554087

**CHURC1->FNTB**	CHURC1	ENSG00000258289.2	29.49959578	30.56789918	28.48154647	28.95235098	28.01475749
**CHURC1->FNTB**	FNTB	ENSG00000257365.2	3.816694181	4.941556596	3.725894548	3.92563567	4.150722432
**CLN6->CALML4**	CALML4	ENSG00000129007.10	4.687672936	4.436549618	4.823664183	4.467116824	4.598959568
**CLN6->CALML4**	CLN6	ENSG00000128973.7	21.37426626	22.75684066	22.43466123	22.29713992	20.79648152
**CNPY2->CS**	CS	ENSG00000062485.12	52.27157097	53.01590061	53.61777626	47.907749	58.36263812
**CNPY2->CS**	CNPY2	ENSG00000257727.1	22.3188971	22.08108367	24.68289803	24.84433729	24.84194893
**COPE->LASS1**	CERS1	ENSG00000223802.2	0.024799596	0.023399348	0.031111688	0.050222758	0.043962274
**COPE->LASS1**	COPE	ENSG00000105669.6	97.06174	96.65078488	101.4763652	103.8010853	104.7536355
**CORO7->PAM16**	PAM16	ENSG00000217930.3	16.61257164	13.56133871	14.50954062	14.85601949	13.79167169
**CORO7->PAM16**	CORO7	ENSG00000262246.1	26.23361989	22.19600499	25.10385143	24.0872176	24.30229396
**COX5A->EDC3**	EDC3	ENSG00000179151.7	15.97126365	17.22856045	15.97889062	15.58101201	15.64561444
**COX5A->EDC3**	COX5A	ENSG00000178741.7	173.6071116	178.4552097	176.2694332	195.8923411	168.0811249
**CTBS->GNG5**	GNG5	ENSG00000174021.6	82.45405822	78.91918376	79.89297998	85.00381096	81.36432123
**CTBS->GNG5**	CTBS	ENSG00000117151.7	5.848730957	5.935160674	5.821543645	5.204300615	6.985368168
**CTSC->RAB38**	RAB38	ENSG00000123892.6	9.613286	8.336437011	9.991831925	10.06216233	5.964564842
**CTSC->RAB38**	CTSC	ENSG00000109861.8	70.35361662	71.44569118	74.62557405	92.12459162	71.16529577
**DDX5->POLG2**	POLG2	ENSG00000256525.1	3.414535894	2.668573292	3.194070301	2.787539582	3.351985032
**DDX5->POLG2**	DDX5	ENSG00000108654.5	180.6971243	156.1679986	177.4357319	160.7129697	171.3901373
**ELAVL1->TIMM44**	TIMM44	ENSG00000104980.2	8.73793433	8.641857382	9.782832806	7.242014505	9.189362011
**ELAVL1->TIMM44**	ELAVL1	ENSG00000066044.8	49.69926751	52.63329931	52.21906297	50.11236663	48.79789117
**ENTPD1->C10orf131**	ENTPD1	ENSG00000138185.11	97.04331488	100.3874839	94.21825482	90.70584376	85.02659447
**ENTPD1->C10orf131**	C10orf131	ENSG00000173088.7	0.320911415	0.23498709	0.688653624	0.264982407	0.304937137
**FAM18B2->CDRT4**	CDRT4	ENSG00000239704.4	0.664369149	0.589553124	0.772501172	0.547304626	1.016598737
**FAM18B2->CDRT4**	FAM18B2	ENSG00000175106.10	1.587252426	1.494444596	1.545950957	1.258719363	1.934665484
**FKBP1A->SDCBP2**	SDCBP2	ENSG00000125775.10	1.185717681	1.465184921	1.223426527	1.121891835	0.897815063
**FKBP1A->SDCBP2**	FKBP1A	ENSG00000088832.10	151.8112761	145.1331044	151.4348642	160.3596183	145.4197875
**GPI->PDCD2L**	GPI	ENSG00000105220.7	94.71795822	97.29952181	103.3726383	111.1396937	98.56551358
**GPI->PDCD2L**	PDCD2L	ENSG00000126249.1	1.810407819	1.475000708	2.04747428	2.401651143	2.044106168
**HACL1->COLQ**	COLQ	ENSG00000206561.6	0.393794426	0.28817373	0.563515516	0.507832011	0.464110863
**HACL1->COLQ**	HACL1	ENSG00000131373.10	13.13739994	11.56013799	12.71274047	13.68510609	12.35554478
**HAUS4->PRMT5**	PRMT5	ENSG00000100462.11	45.62853494	43.05536373	46.29080424	43.65171658	48.13785332
**HAUS4->PRMT5**	HAUS4	ENSG00000092036.12	30.10984732	32.96760066	32.6194606	32.54630134	27.74455814
**HMSD->SERPINB8**	HMSD	ENSG00000221887.4	10.44849236	11.10453329	10.01069552	11.04479373	10.36009139
**HMSD->SERPINB8**	SERPINB8	ENSG00000166401.9	4.491313489	4.797049213	4.195911613	4.102121143	4.432158737
**HSPE1->MOBKL3**	HSPE1	ENSG00000115541.6	91.0937302	66.18157811	89.32332791	78.81429511	85.89132665
**HSPE1->MOBKL3**	MOB4	ENSG00000115540.10	16.86638544	14.36052404	16.97855692	15.34599871	15.41449714
**IFNAR2->IL10RB**	IFNAR2	ENSG00000159110.14	30.13825871	29.46107136	30.30952872	28.69344234	30.57395911
**IFNAR2->IL10RB**	IL10RB	ENSG00000243646.3	21.9458002	24.70549733	21.98620929	24.1207587	24.02447018
**IFRD1->C7orf53**	IFRD1	ENSG00000006652.8	13.73716048	11.14045135	13.42208761	11.79511121	12.98334319
**IFRD1->C7orf53**	C7orf53	ENSG00000181016.4	0.540997298	0.555350596	0.537467226	0.386363912	0.484112442
**ISY1->RAB43**	RAB43	ENSG00000172780.12	1.631622766	1.952500281	1.523514226	1.606611484	1.276206632
**ISY1->RAB43**	ISY1	ENSG00000240682.5	28.08584006	28.56063983	27.68435559	28.20269071	26.57386575
**JAK3->INSL3**	INSL3	ENSG00000248099.2	0.520239713	0.482505933	0.472092215	0.498491615	0.686749474
**JAK3->INSL3**	JAK3	ENSG00000105639.13	18.09790718	17.76316657	17.43255241	17.34182145	19.86869315
**KIAA0101->CSNK1G1**	CSNK1G1	ENSG00000169118.9	3.591358819	4.175923348	3.203740914	3.159449791	3.695531095
**KIAA0101->CSNK1G1**	KIAA0101	ENSG00000166803.5	65.10393533	67.88663004	64.46905661	69.69149123	59.2100858
**KIAA0494->ATPAF1**	ATPAF1	ENSG00000123472.8	32.35419051	34.71451294	35.26358035	38.17696593	31.72057255
**KIAA0494->ATPAF1**	KIAA0494	ENSG00000159658.5	16.56303957	19.13207283	16.02096187	15.20797152	15.8357
**LMAN2->MXD3**	MXD3	ENSG00000213347.5	16.06503674	15.66238597	14.97050694	16.20341408	16.00549326
**LMAN2->MXD3**	LMAN2	ENSG00000169223.9	62.59427862	60.78904744	67.91960418	57.93592569	62.51516754
**LRRC33->PIGX**	LRRC33	ENSG00000174004.5	10.9743448	11.93340037	11.93187861	9.971475429	11.64545187
**LRRC33->PIGX**	PIGX	ENSG00000163964.9	38.16328966	36.13192967	34.62720608	33.26104333	35.59069702
**LSP1->TNNT3**	LSP1	ENSG00000130592.9	227.640345	262.5865592	240.8769295	292.7315256	264.8893665
**LSP1->TNNT3**	TNNT3	ENSG00000130595.11	0.977944968	0.941297112	0.815124538	0.874275495	1.538645368
**MED8->ELOVL1**	ELOVL1	ENSG00000066322.7	38.88604965	42.71096529	39.23992433	37.98503959	37.40454725
**MED8->ELOVL1**	MED8	ENSG00000159479.12	15.69836064	17.42704034	16.48201184	15.03327925	17.33903559
**METTL10->FAM53B**	FAM53B	ENSG00000189319.9	2.615862606	3.165027764	2.885907	2.86194944	3.012508168
**METTL10->FAM53B**	METTL10	ENSG00000203791.6	22.51393372	22.31454193	22.51328119	22.91206757	22.15960552
**METTL21B->TSFM**	METTL21B	ENSG00000123427.11	2.139612319	1.955499764	2.351806527	2.114925022	2.633137632
**METTL21B->TSFM**	TSFM	ENSG00000123297.11	15.02415877	16.17295261	17.03567467	15.61530492	14.01766413
**NDUFA13->YJEFN3**	NDUFA13	ENSG00000186010.9	102.3347326	101.7854121	105.0337302	120.0998208	102.0109932
**NDUFA13->YJEFN3**	YJEFN3	ENSG00000250067.3	0.569091457	0.588441382	0.586276194	0.613525967	0.587202442
**NDUFB8->SEC31B**	SEC31B	ENSG00000075826.11	11.82822277	8.360808876	10.16208614	10.47374534	10.77665807
**NDUFB8->SEC31B**	NDUFB8	ENSG00000166136.10	97.2061706	91.73054164	101.2745246	101.7006544	92.39713079
**NRXN1->EIF2AK2**	EIF2AK2	ENSG00000055332.10	23.56409428	25.34402774	21.86252872	19.10333359	20.80509737
**NRXN1->EIF2AK2**	NRXN1	ENSG00000179915.14	0.025474383	0.030741933	0.023074409	0.062749275	0.053395495
**NSUN4->FAAH**	NSUN4	ENSG00000117481.5	13.65531738	16.96921907	14.61061995	13.14612344	13.77807953
**NSUN4->FAAH**	FAAH	ENSG00000117480.10	0.777518138	0.534342427	0.889519538	0.692011121	0.8215024
**PEX26->TUBA8**	PEX26	ENSG00000215193.5	18.36108251	18.34654311	16.99203158	16.69669212	16.50116769
**PEX26->TUBA8**	TUBA8	ENSG00000183785.9	9.743676415	6.694677685	7.205884688	6.246841538	6.894373621
**PFKFB4->SHISA5**	SHISA5	ENSG00000164054.10	71.96781456	84.21802775	66.26101134	59.51278299	57.37454387
**PFKFB4->SHISA5**	PFKFB4	ENSG00000114268.7	45.9500127	51.00611574	50.52991553	49.79434949	45.64518295
**PKHD1L1->EBAG9**	PKHD1L1	ENSG00000205038.7	2.602326372	3.049869034	2.488639301	2.395403374	2.705607768
**PKHD1L1->EBAG9**	EBAG9	ENSG00000147654.10	7.557159574	5.36721182	6.898129882	6.393031626	7.770343747
**PLEKHO2->ANKDD1A**	PLEKHO2	ENSG00000241839.4	13.17010477	18.1881491	14.42617317	14.2156483	13.46940696
**PLEKHO2->ANKDD1A**	ANKDD1A	ENSG00000166839.11	6.202863968	2.619612685	4.591406419	4.898110516	4.557899453
**POLA2->CDC42EP2**	POLA2	ENSG00000014138.4	35.49797926	33.52681615	37.33786666	35.42313387	35.27246092

**POLA2->CDC42EP2**	CDC42EP2	ENSG00000149798.3	0.631257543	0.336539124	0.396313151	0.371217231	0.287204053
**PPRC1->NOLC1**	PPRC1	ENSG00000148840.6	14.97833789	15.31191181	15.92749928	12.6933593	14.68553951
**PPRC1->NOLC1**	NOLC1	ENSG00000166197.11	54.44655366	51.70391227	55.59346039	50.48721347	51.74183652
**PRH1->PRR4**	PRR4	ENSG00000111215.5	6.132140883	5.317343225	5.15392029	5.319651407	5.032303558
**PRH1->PRR4**	PRH1	ENSG00000231887.2	0.051987191	0.035595742	0.040384194	0.043061011	0.047493421
**PRIM1->NACA**	NACA	ENSG00000196531.6	378.0024264	315.8285422	367.0260718	393.6459119	367.7726556
**PRIM1->NACA**	PRIM1	ENSG00000198056.6	55.84819487	41.22398626	42.54310005	46.29436356	48.87769654
**PRKAA1->TTC33**	TTC33	ENSG00000113638.8	6.822513979	7.282540191	6.730802376	6.159106593	6.902695495
**PRKAA1->TTC33**	PRKAA1	ENSG00000132356.7	13.18766986	14.05829781	12.89184319	10.8582143	12.54938963
**PRR11->C17orf71**	PRR11	ENSG00000068489.7	12.67610985	21.61786051	13.90173472	12.39064007	14.68808017
**PRR11->C17orf71**	SMG8	ENSG00000167447.7	6.081027053	6.820464888	6.088899581	5.4061	6.033446432
**PRR13->PCBP2**	PRR13	ENSG00000205352.6	109.1367621	108.0161139	111.5153818	113.2196019	105.1048525
**PRR13->PCBP2**	PCBP2	ENSG00000197111.8	139.4203555	157.3791197	141.3378427	150.3910683	145.8304348
**PXMP2->PGAM5**	PXMP2	ENSG00000176894.5	20.02667607	20.1695346	22.01420606	22.734633	24.27209135
**PXMP2->PGAM5**	PGAM5	ENSG00000247077.2	12.31697193	13.75460031	13.92640112	13.29002975	11.72121974
**RBM14->RBM4**	RBM14	ENSG00000239306.3	30.74817491	32.73529203	32.95463278	31.28421813	31.14510042
**RBM14->RBM4**	RBM4	ENSG00000173933.13	65.54143519	68.1221944	63.32697491	64.58052941	60.35339796
**RNASET2->RPS6KA2**	RPS6KA2	ENSG00000071242.7	2.760328564	2.124262697	2.562097247	1.715952451	2.131388211
**RNASET2->RPS6KA2**	RNASET2	ENSG00000026297.10	123.6780107	122.8803255	124.0424791	109.6018191	94.03306868
**RRM2->C2orf48**	RRM2	ENSG00000171848.7	163.2499859	185.274968	190.4112143	163.4417439	157.9946831
**RRM2->C2orf48**	C2orf48	ENSG00000163009.4	0.032690128	0.037639539	0.049947194	0.023433374	0.0246288
**S1PR2->DNMT1**	DNMT1	ENSG00000130816.8	13.4169065	16.00563763	13.78435169	13.40846225	12.08936049
**S1PR2->DNMT1**	S1PR2	ENSG00000175898.3	8.434811181	10.97790184	8.979750645	10.12715192	9.923880474
**SDHAF2->C11orf66**	SDHAF2	ENSG00000167985.2	24.43844747	23.1344353	24.12946994	23.30089876	23.08944715
**SDHAF2->C11orf66**	PPP1R32	ENSG00000162148.5	1.511087574	0.869939517	1.255444548	1.036493198	1.363887505
**SDHD->TEX12**	SDHD	ENSG00000204370.4	44.91188222	44.09153081	44.7732534	44.05328969	44.47096317
**SDHD->TEX12**	TEX12	ENSG00000150783.5	0.237343309	0.035136685	0.087332882	0.117434934	0.061957979
**SLC35A3->HIAT1**	SLC35A3	ENSG00000117620.7	5.225617511	5.281971191	5.527833108	4.801312582	5.343578632
**SLC35A3->HIAT1**	HIAT1	ENSG00000156875.9	18.32397881	18.02828407	17.48654761	18.49502791	17.90269295
**SLC39A1->CRTC2**	CRTC2	ENSG00000160741.11	29.465655	31.93469482	28.03187412	28.4621183	30.76706087
**SLC39A1->CRTC2**	SLC39A1	ENSG00000143570.12	42.8261905	42.26228497	42.54993456	43.08067718	41.43536616
**SLC43A3->PRG2**	PRG2	ENSG00000186652.5	0.79053966	0.672652101	0.614011226	0.346475352	0.535416011
**SLC43A3->PRG2**	SLC43A3	ENSG00000134802.12	90.6021067	87.2709679	89.15472418	78.47277627	87.88464064
**SMG1->ARL6IP1**	ARL6IP1	ENSG00000170540.9	38.90744906	38.42395615	35.37175469	34.78713248	36.90102269
**SMG1->ARL6IP1**	SMG1	ENSG00000157106.12	6.538458691	8.204418674	5.995910387	4.852424121	6.370255947
**SNTB2->VPS4A**	SNTB2	ENSG00000168807.11	2.474970777	2.611984607	2.97143071	2.198533681	2.393718105
**SNTB2->VPS4A**	VPS4A	ENSG00000132612.13	22.16466353	22.21853804	22.56465816	21.37079852	21.55333633
**SUMO2->HN1**	HN1	ENSG00000189159.9	124.016791	126.9551137	136.224488	133.8085991	121.2384035
**SUMO2->HN1**	SUMO2	ENSG00000188612.6	103.7767504	96.12969274	102.6198414	102.7107278	100.3119416
**SYNJ2BP->COX16**	COX16	ENSG00000133983.9	32.83687428	27.4407762	31.15581724	30.77746836	30.06204924
**SYNJ2BP->COX16**	SYNJ2BP	ENSG00000213463.3	6.668464138	6.648277303	5.695307505	6.224736648	5.424950589
**TAGLN2->CCDC19**	CCDC19	ENSG00000213085.5	0.49447266	0.492843775	0.483313247	0.458140275	0.462106053
**TAGLN2->CCDC19**	TAGLN2	ENSG00000158710.10	237.0772916	193.2351802	228.0792703	235.6893281	225.6838114
**TAP2->HLA-DOB**	HLA-DOB	ENSG00000241106.2	17.11854495	20.06486801	18.66704585	30.73758727	20.90948188
**TAP2->HLA-DOB**	TAP2	ENSG00000204267.8	40.82895891	46.28916572	42.46433861	30.24132603	37.26369034
**TMBIM4->LLPH**	LLPH	ENSG00000139233.2	4.930487787	3.394681292	5.137487505	4.484556484	4.647875021
**TMBIM4->LLPH**	TMBIM4	ENSG00000155957.10	36.57890733	31.59536555	36.55538615	33.51178535	35.19492414
**TNFAIP8L2->SCNM1**	TNFAIP8L2	ENSG00000163154.5	9.27864284	7.576078461	9.024370118	8.604165022	6.748807021
**TNFAIP8L2->SCNM1**	SCNM1	ENSG00000163156.6	16.26255895	15.92181161	16.0542534	15.66787104	15.48852525
**TOMM5->FBXO10**	FBXO10	ENSG00000147912.8	5.143780809	5.550988764	4.241497688	5.623400934	4.959743926
**TOMM5->FBXO10**	TOMM5	ENSG00000175768.8	71.46876095	68.63434344	68.80053262	71.00461126	66.27943279
**TOPORS->DDX58**	DDX58	ENSG00000107201.4	11.2570904	9.117292236	9.426542538	6.659911099	7.029413726
**TOPORS->DDX58**	TOPORS	ENSG00000197579.3	5.622489596	5.300081764	5.899320387	5.506137363	5.898674684
**TPD52L2->DNAJC5**	TPD52L2	ENSG00000101150.11	25.85194889	26.63020128	26.47693635	24.46498023	25.24630527
**TPD52L2->DNAJC5**	DNAJC5	ENSG00000101152.6	10.41210614	11.66717385	9.375657806	8.590418868	9.182412842
**TSC22D4->C7orf61**	C7orf61	ENSG00000185955.4	0.235787968	0.292807371	0.211122946	0.261146934	0.220979095
**TSC22D4->C7orf61**	TSC22D4	ENSG00000166925.4	21.70328087	22.76395848	21.80779685	20.76472401	19.54516563
**TSTD1->F11R**	F11R	ENSG00000158769.11	20.84950255	17.34564073	18.31992427	19.91916948	18.42823865
**TSTD1->F11R**	TSTD1	ENSG00000215845.6	35.1029513	27.73719366	32.74639628	39.00713815	34.18505124
**UBA2->WTIP**	UBA2	ENSG00000126261.5	47.20897221	47.57220797	49.10398999	48.48336032	47.87353502
**UBA2->WTIP**	WTIP	ENSG00000142279.6	0.031896723	0.00180264	0.035569817	0.054626659	0.027588747
**UBE2J1->GABRR2**	GABRR2	ENSG00000111886.8	0.084018032	0.090059202	0.153752344	0.057646879	0.084693937
**UBE2J1->GABRR2**	UBE2J1	ENSG00000198833.5	89.99867701	100.7781554	95.50390467	75.11159311	97.88156763
**UBE2J2->FAM132A**	FAM132A	ENSG00000184163.3	1.227618606	1.129619315	1.333126806	0.878630758	1.519782358
**UBE2J2->FAM132A**	UBE2J2	ENSG00000160087.16	22.806382	22.27736915	23.76680509	23.95978949	22.73817327
**UCHL3->LMO7**	UCHL3	ENSG00000118939.10	12.26525898	10.26847754	8.455602419	12.09196027	9.657709611
**UCHL3->LMO7**	LMO7	ENSG00000136153.13	18.16348514	13.30850847	8.712026452	20.48728129	13.65921448
**UQCRQ->LEAP2**	UQCRQ	ENSG00000164405.6	120.9763599	118.4041961	121.2431535	125.2558714	118.6619284
**UQCRQ->LEAP2**	LEAP2	ENSG00000164406.7	1.359685957	1.322331831	1.194955688	1.25603167	1.118377705
**VKORC1->PRSS53**	PRSS53	ENSG00000151006.7	1.053399691	0.985587921	0.926315086	0.832715945	0.859518358
**VKORC1->PRSS53**	VKORC1	ENSG00000167397.10	36.04180953	33.08685003	36.36549349	39.79408975	36.13688757
**ZNF343->SNRPB**	SNRPB	ENSG00000125835.12	124.0939238	133.0064607	138.3087852	128.6012789	122.7281264
**ZNF343->SNRPB**	ZNF343	ENSG00000088876.7	3.634856521	3.705674034	3.388533796	3.232406956	3.567132442

Table S6

Chimera	Transcript IDs gene1	Exon number gene1	Exon counts gene1	Exon status gene1	Distance from exon ends for gene1 *	Transcript IDs gene2	Exon number gene2	Exon counts gene2	Exon status gene2	Distance from exon ends for gene2*
**AP5S1->MAVS**	ENST00000246041.2,	2,	3,	exon,	0	ENST00000356687.4,	2,	4,	5UTR,	0
	ENST00000379567.2,	2,	3,	exon,		ENST00000416600.2,	2,	6,	5UTR,	
	ENST00000379573.2,	2,	3,	exon,		ENST00000428216.2	2	7	5UTR	
	ENST00000455742.1	2	3	exon						
**ARHGAP19->SLIT1**	ENST00000316676.8,	11,	12,	exon,	0	ENST00000266058.4,	2,	37,	exon,	0
	ENST00000355366.5,	11,	12,	exon,		ENST00000314867.5,	2,	22,	exon,	
	ENST00000358308.3,	10,	11,	exon,		ENST00000371041.3,	2,	12,	exon,	
	ENST00000358531.4,	11,	12,	exon,		ENST00000371054.1,	2,	36,	exon,	
	ENST00000371027.1,	11,	12,	exon,		ENST00000371057.1,	2,	36,	exon,	
	ENST00000393817.2,	9,	10,	exon,		ENST00000371070.4,	2,	37,	exon,	
	ENST00000453547.2,	11,	47,	exon,		ENST00000456008.1	2	12	exon	
	ENST00000479633.1,	9,	10,	exon,						
	ENST00000487035.1,	6,	7,	exon,						
	ENST00000492211.1	12	13	exon						
**C11orf48->INTS5**	ENST00000354588.3,	6,	7,	exon,	0	ENST00000330574.2	2	2	exon	0
	ENST00000431002.2,	4,	5,	exon,						
	ENST00000524958.1,	3,	4,	exon,						
	ENST00000532208.1,	6,	7,	exon,						
	ENST00000532786.1	2	3	exon						
**C7orf55->LUC7L2**	ENST00000297534.6,	2,	2,	exon,	84,	ENST00000263545.6,	2,	10,	exon,	0
	ENST00000468383.1,	2,	2,	exon,	174,	ENST00000354926.4,	2,	10,	exon,	
	ENST00000481123.1,	2,	2,	exon,	169,	ENST00000448820.1,	3,	6,	exon,	
	ENST00000482181.1,	2,	2,	exon,	156,	ENST00000456182.1,	3,	11,	exon,	
	ENST00000488886.1	3	3	exon	174	ENST00000541170.1,	3,	11,	exon,	
						ENST00000541515.1,	3,	11,	exon,	
						ENST00000545899.1	3	11	exon	
**CCL22->CX3CL1**	ENST00000219235.4	2	3	exon	0	ENST00000006053.6	2	3	exon	0
**CENPE->BDH2**	ENST00000265148.3,	48,	49,	exon,	0	ENST00000296424.4,	2,	10,	5UTR,	0
	ENST00000380026.3,	46,	47,	exon,		ENST00000464039.1,	3,	10,	exon,	
	ENST00000394771.1	49	50	exon		ENST00000475058.1,	2,	10,	exon,	
						ENST00000492366.1,	2,	9,	exon,	
						ENST00000504285.1,	3,	7,	5UTR,	
						ENST00000506521.1,	2,	6,	5UTR,	
						ENST00000509245.1,	3,	6,	5UTR,	
						ENST00000511354.1,	2,	7,	5UTR,	
						ENST00000513518.1	2	4	exon	
**CHURC1->FNTB**	ENST00000359118.2,	2,	4,	exon,	0	ENST00000246166.2,	2,	12,	exon,	0
	ENST00000547625.1,	1,	3,	exon,		ENST00000555372.1,	2,	5,	exon,	
	ENST00000548752.2,	2,	3,	exon,		ENST00000555742.1	2	5	exon	
	ENST00000549115.1,	2,	4,	exon,						
	ENST00000551093.1,	2,	4,	exon,						
	ENST00000551947.1,	2,	3,	exon,						
	ENST00000552002.1	2	4	exon						
**CLN6->CALML4**	ENST00000249806.5,	1,	7,	exon,	0	ENST00000354678.3,	2,	2,	exon,	0
	ENST00000418702.2,	1,	5,	5UTR,		ENST00000395463.2,	3,	4,	3UTR,	
	ENST00000564752.1,	1,	7,	exon,		ENST00000448060.2,	3,	4,	exon,	
	ENST00000565471.1,	1,	3,	exon,		ENST00000467889.1,	4,	5,	exon,	
	ENST00000566347.1,	1,	6,	exon,		ENST00000478113.1,	2,	3,	exon,	
	ENST00000567060.1,	1,	5,	exon,		ENST00000540479.1	3	4	exon	
	ENST00000569336.1	1	3	exon						
**CNPY2->CS**	ENST00000273308.4,	3,	6,	exon,	0	ENST00000351328.3,	2,	11,	exon,	0
	ENST00000551276.1,	2,	3,	exon,		ENST00000542324.2,	3,	12,	exon,	
	ENST00000551286.1,	2,	4,	exon,		ENST00000546554.1,	2,	5,	exon,	
	ENST00000551475.1,	3,	5,	exon,		ENST00000546930.1,	2,	5,	exon,	

	ENST00000551720.1,	3,	4,	exon,		ENST00000547283.1,	2,	4,	exon,	
	ENST00000553191.1	1	4	exon		ENST00000547298.1,	2,	6,	5UTR,	
						ENST00000548041.1,	2,	6,	exon,	
						ENST00000548567.1,	3,	12,	5UTR,	
						ENST00000548849.1,	2,	7,	exon,	
						ENST00000549143.1,	2,	11,	exon,	
						ENST00000550655.1,	3,	7,	exon,	
						ENST00000550996.1,	2,	2,	exon,	
						ENST00000551137.1,	4,	8,	5UTR,	
						ENST00000551253.1,	2,	7,	5UTR,	
						ENST00000551430.1,	3,	6,	5UTR,	
						ENST00000551441.1,	3,	3,	exon,	
						ENST00000551473.1,	3,	7,	5UTR,	
						ENST00000551968.1,	2,	6,	exon,	
						ENST00000552222.1,	2,	6,	exon,	
						ENST00000552688.1	2	5	exon	
**COPE->CERS1**	ENST00000262812.3,	9,	10,	exon,	0	ENST00000427170.2,	3,	8,	exon,	0
	ENST00000349893.3,	8,	9,	exon,		ENST00000429504.1,	3,	6,	exon,	
	ENST00000351079.3,	8,	9,	exon,		ENST00000542296.1	3	6	exon	
	ENST00000538245.1	12	13	exon						
**CORO7->PAM16**	ENST00000251166.4,	27,	28,	exon,	0	ENST00000318059.3,	2,	5,	exon,	0
	ENST00000537233.2,	27,	28,	exon,		ENST00000571819.1,	2,	3,	exon,	
	ENST00000539968.1,	27,	28,	exon,		ENST00000571941.1,	3,	6,	exon,	
	ENST00000571227.1,	28,	29,	3UTR,		ENST00000571986.1,	2,	5,	exon,	
	ENST00000572274.1,	5,	7,	exon,		ENST00000573553.1,	2,	5,	exon,	
	ENST00000572467.1,	27,	31,	exon,		ENST00000573614.1,	2,	5,	exon,	
	ENST00000574025.1,	25,	26,	exon,		ENST00000575636.1,	2,	5,	exon,	
	ENST00000575334.1,	29,	33,	3UTR,		ENST00000575848.1,	2,	5,	exon,	
	ENST00000576637.1	5	6	3UTR		ENST00000576217.1,	3,	6,	exon,	
						ENST00000577031.1	2	5	exon	
**COX5A->EDC3**	ENST00000322347.6,	1,	5,	exon,	0	ENST00000315127.4,	5,	7,	exon,	0
	ENST00000562233.1,	1,	3,	exon,		ENST00000426797.3,	8,	10,	exon,	
	ENST00000564811.1,	1,	4,	exon,		ENST00000562174.1,	2,	3,	exon,	
	ENST00000567270.1,	1,	4,	exon,		ENST00000562974.1,	2,	2,	exon,	
	ENST00000568783.1	1	4	exon		ENST00000565602.1,	4,	6,	3UTR,	
						ENST00000566219.1,	3,	4,	exon,	
						ENST00000568176.1	6	8	exon	
**CTBS->GNG5**	ENST00000370625.1,	6,	7,	3UTR,	0	ENST00000370641.3,	2,	3,	exon,	0
	ENST00000370630.4,	6,	7,	exon,		ENST00000370645.4,	3,	4,	exon,	
	ENST00000465118.2,	5,	6,	3UTR,		ENST00000487806.1	2	3	exon	
	ENST00000477677.1	5	6	exon						
**CTSC->RAB38**	ENST00000227266.4,	5,	7,	exon,	0	ENST00000243662.5,	2,	3,	exon,	0
	ENST00000393302.3,	6,	8,	exon,		ENST00000526372.1	2	3	exon	
	ENST00000527018.1	5	6	exon						
**DDX5->POLG2**	ENST00000225792.4,	13,	14,	exon,	0	ENST00000539111.1	2	8	exon	0
	ENST00000450599.1,	11,	12,	exon,						
	ENST00000540698.1	12	13	exon						
**ELAVL1->TIMM44**	ENST00000351593.5,	4,	6,	exon,	0	ENST00000270538.2	2	13	exon	0
	ENST00000407627.1	4	6	exon						
**ENTPD1->C10orf131**	ENST00000371203.5,	8,	9,	exon,	0	ENST00000423344.2,	2,	8,	5UTR,	0
	ENST00000371205.4,	9,	10,	exon,		ENST00000491114.1	3	7	exon	
	ENST00000371207.3,	9,	10,	exon,						
	ENST00000453258.2,	9,	10,	exon,						
	ENST00000466637.1,	1,	3,	exon,						
	ENST00000539125.1,	7,	8,	exon,						
	ENST00000543964.1	8	9	exon						
**FAM18B2->CDRT4**	ENST00000225576.3,	5,	6,	exon,	0	ENST00000312177.5,	3,	4,	5UTR,	0
	ENST00000419890.2,	5,	6,	exon,		ENST00000519864.1,	3,	4,	5UTR,	
	ENST00000428082.1,	5,	6,	exon,		ENST00000520956.1,	4,	6,	5UTR,	

	ENST00000438826.2,	5,	7,	exon,		ENST00000524205.1	2	4	5UTR	
	ENST00000518321.1,	5,	6,	exon,						
	ENST00000519970.1,	4,	7,	exon,						
	ENST00000521179.1,	4,	6,	3UTR,						
	ENST00000523573.1	5	7	3UTR						
FKBP1A->SDCBP2	ENST00000381715.1,	1,	3,	exon,	0	ENST00000339987.3,	2,	9,	5UTR,	0
	ENST00000381719.3,	2,	4,	exon,		ENST00000360779.3,	2,	9,	5UTR,	
	ENST00000381724.3,	1,	4,	exon,		ENST00000381812.1	2	9	5UTR	
	ENST00000400137.4	2	5	exon						
GPI->PDCD2L	ENST00000356487.3,	14,	18,	exon,	0	ENST00000246535.1	2	7	exon	0
	ENST00000415930.2	14	18	exon						
HACL1->COLQ	ENST00000321169.5,	16,	17,	exon,	0	ENST00000383781.3,	2,	17,	exon,	0
	ENST00000383779.4,	14,	15,	3UTR,		ENST00000383785.2,	2,	18,	exon,	
	ENST00000422591.1,	14,	14,	3UTR,		ENST00000383786.3,	2,	16,	exon,	
	ENST00000435217.2,	8,	9,	exon,		ENST00000383787.2,	2,	16,	exon,	
	ENST00000451445.2,	13,	14,	exon,		ENST00000383788.3,	2,	17,	exon,	
	ENST00000456194.2,	15,	16,	exon,		ENST00000420589.1,	2,	18,	exon,	
	ENST00000457447.2	14	15	exon		ENST00000430319.1,	2,	11,	exon,	
						ENST00000435459.2,	2,	17,	exon,	
						ENST00000454772.1	2	17	exon	
HAUS4->PRMT5	ENST00000206474.7,	9,	10,	exon,	0	ENST00000216350.8,	2,	16,	exon,	0
	ENST00000342454.8,	8,	9,	exon,		ENST00000324366.8,	2,	17,	exon,	
	ENST00000347758.2,	6,	7,	exon,		ENST00000397440.4,	2,	13,	exon,	
	ENST00000397409.4,	6,	7,	exon,		ENST00000397441.2,	2,	17,	exon,	
	ENST00000490506.1,	8,	9,	exon,		ENST00000421938.2,	2,	5,	exon,	
	ENST00000541587.1,	9,	10,	exon,		ENST00000553550.1,	2,	5,	exon,	
	ENST00000553859.1,	5,	6,	3UTR,		ENST00000553641.1,	2,	7,	exon,	
	ENST00000554446.1,	6,	7,	exon,		ENST00000553897.1,	2,	16,	exon,	
	ENST00000555367.1,	8,	9,	exon,		ENST00000554716.1,	2,	5,	exon,	
	ENST00000555986.1,	8,	9,	exon,		ENST00000554867.1,	2,	6,	exon,	
	ENST00000556421.1	2	3	exon		ENST00000554910.1,	2,	7,	5UTR,	
						ENST00000556032.1,	2,	3,	exon,	
						ENST00000556426.1,	2,	2,	exon,	
						ENST00000557015.1	2	2	exon	
HMSD->SERPINB8	ENST00000481726.1,	3,	6,	exon,	0	ENST00000353706.2,	2,	7,	5UTR,	0
	ENST00000498680.1	1	2	exon		ENST00000397985.2,	2,	7,	5UTR,	
						ENST00000397988.3,	2,	7,	5UTR,	
						ENST00000441827.1,	2,	5,	5UTR,	
						ENST00000448851.1	2	4	5UTR	
HSPE1->MOBKL3	ENST00000233893.5,	3,	4,	exon,	0	ENST00000233892.4,	2,	8,	5UTR,	0
	ENST00000409729.1,	2,	3,	exon,		ENST00000323303.4,	2,	8,	exon,	
	ENST00000465573.1	2	3	exon		ENST00000409360.1,	2,	8,	5UTR,	
						ENST00000417097.1	2	7	exon	
IFNAR2->IL10RB	ENST00000342101.3,	7,	8,	exon,	0	ENST00000290200.2,	2,	7,	exon,	0
	ENST00000342136.4,	7,	9,	exon,		ENST00000422891.1,	2,	6,	exon,	
	ENST00000382238.2,	8,	10,	3UTR,		ENST00000539894.1	2	8	exon	
	ENST00000382241.3,	7,	9,	exon,						
	ENST00000382264.3,	7,	9,	exon,						
	ENST00000404220.2,	7,	9,	exon,						
	ENST00000413881.1,	5,	6,	exon,						
	ENST00000417007.1,	3,	5,	3UTR,						
	ENST00000443073.1	5	7	exon						
IFRD1->C7orf53	ENST00000005558.4,	12,	13,	exon,	0	ENST00000312849.3,	2,	4,	5UTR,	0
	ENST00000403825.3,	11,	12,	exon,		ENST00000429049.1,	2,	3,	5UTR,	
	ENST00000462155.2,	3,	4,	exon,		ENST00000439068.2,	2,	4,	5UTR,	
	ENST00000470441.1,	3,	3,	exon,		ENST00000455302.1	3	6	5UTR	
	ENST00000489994.1,	3,	4,	exon,						
	ENST00000535603.1,	11,	12,	exon,						
	ENST00000536259.1	10	10	exon						

**ISY1->RAB43**	ENST00000273541.8, ENST00000393295.3, ENST00000485703.1, ENST00000496163.1	9, 8, 9, 3	12, 11, 10, 5	exon, exon, 3UTR, exon	0	ENST00000315150.5, ENST00000393304.1, ENST00000393305.1, ENST00000393307.1, ENST00000393308.1, ENST00000457077.1, ENST00000476465.1	2, 3, 3, 3, 3, 3, 2	3, 4, 4, 4, 4, 3, 4	exon, exon, exon, exon, exon, exon, exon	0
**JAK3->INSL3**	ENST00000458235.1, ENST00000527670.1	23, 22	24, 23	exon, exon	0	ENST00000317306.6, ENST00000379695.4	2, 3	2, 3	exon, exon	0
**KIAA0101->CSNK1G1**	ENST00000300035.4, ENST00000558008.1, ENST00000558043.1, ENST00000559519.1, ENST00000560234.1	3, 3, 2, 2, 3	4, 5, 3, 3, 4	exon, exon, exon, exon, 3UTR	0	ENST00000303032.6, ENST00000303052.7, ENST00000447727.2	2, 2, 3	11, 12, 12	5UTR, 5UTR, 5UTR	0
**KIAA0494->ATPAF1**	ENST00000371933.3, ENST00000479745.1, ENST00000481623.1, ENST00000487741.1, ENST00000544071.1	10, 4, 3, 5, 9	11, 5, 4, 6, 11	exon, exon, exon, exon, exon	0	ENST00000329231.4, ENST00000371937.4, ENST00000460928.1, ENST00000474020.1, ENST00000487193.1, ENST00000525633.1, ENST00000529214.1, ENST00000532925.1, ENST00000542495.1, ENST00000574428.1, ENST00000576409.1	2, 2, 2, 2, 2, 2, 2, 2, 2, 2, 2	7, 9, 2, 6, 7, 4, 10, 9, 10, 7, 9	exon, exon, exon, exon, exon, exon, exon, exon, 5UTR, exon, exon	0
**LMAN2->MXD3**	ENST00000303127.7, ENST00000504071.1, ENST00000514458.1, ENST00000515209.1, ENST00000539488.1	7, 1, 5, 7, 8	8, 2, 6, 8, 9	exon, exon, exon, exon, exon	0	ENST00000303165.5, ENST00000423571.2, ENST00000427908.2, ENST00000439742.2, ENST00000502529.1, ENST00000503473.1, ENST00000503782.1, ENST00000513063.1	2, 2, 2, 2, 2, 2, 2, 3	5, 5, 6, 6, 4, 11, 4, 7	exon, exon, exon, exon, exon, exon, exon, exon	0
**LRRC33->PIGX**	ENST00000328557.4	2	3	exon	0	ENST00000296333.5, ENST00000314118.4, ENST00000392391.3, ENST00000415832.1, ENST00000421265.1, ENST00000426755.1, ENST00000451319.1, ENST00000453218.2, ENST00000457284.1, ENST00000495440.1, ENST00000541663.1	2, 2, 2, 2, 2, 3, 2, 2, 2, 2, 2	7, 6, 6, 8, 4, 6, 4, 7, 5, 4, 8	exon, 5UTR, exon, 5UTR, 5UTR, 5UTR, 5UTR, exon, exon, exon, 5UTR	0
**LSP1->TNNT3**	ENST00000311604.3, ENST00000381775.1, ENST00000405957.2, ENST00000406638.2, ENST00000472974.1, ENST00000485341.1	10, 11, 10, 10, 9, 9	11, 12, 11, 11, 9, 10	3UTR, 3UTR, 3UTR, 3UTR, exon, exon	0	ENST00000278317.6, ENST00000344578.4, ENST00000360603.3, ENST00000381548.3, ENST00000381549.3, ENST00000381557.2, ENST00000381558.1, ENST00000381561.4, ENST00000381563.4, ENST00000381579.3, ENST00000381589.3, ENST00000397309.3, ENST00000453458.1, ENST00000544980.1	2, 2, 2, 2, 2, 2, 2, 2, 2, 2, 2, 2, 2, 2	16, 14, 15, 16, 15, 14, 15, 16, 17, 15, 16, 17, 11, 10	5UTR, 5UTR, 5UTR, 5UTR, 5UTR, 5UTR, 5UTR, 5UTR, 5UTR, 5UTR, 5UTR, 5UTR, 5UTR, 5UTR	0
**MED8->ELOVL1**	ENST00000290663.6	7	8	exon	0	ENST00000372458.3,	2,	8,	5UTR,	0

						ENST00000413844.2,	2,	7,	5UTR,	
						ENST00000464204.1,	2,	8,	exon,	
						ENST00000465321.1,	3,	7,	exon,	
						ENST00000470769.1,	2,	8,	exon,	
						ENST00000470968.1,	2,	6,	exon,	
						ENST00000479439.1,	2,	6,	exon,	
						ENST00000479686.1,	2,	5,	exon,	
						ENST00000482302.1,	3,	9,	exon,	
						ENST00000487209.1,	2,	7,	exon,	
						ENST00000496932.1,	2,	4,	exon,	
						ENST00000497050.1,	2,	8,	exon,	
						ENST00000497569.1	2	8	exon	
**METTL10->FAM53B**	ENST00000495711.1	1	2	exon	1	ENST00000280780.6,	2,	5,	5UTR,	1
						ENST00000337318.3,	2,	5,	5UTR,	
						ENST00000392754.3	2	5	5UTR	
**METTL21B->TSFM**	ENST00000300209.8,	2,	3,	exon,	0	ENST00000323833.8,	2,	7,	exon,	0
	ENST00000333012.5,	2,	4,	exon,		ENST00000350762.5,	2,	8,	5UTR,	
	ENST00000548256.1,	2,	4,	exon,		ENST00000417094.1,	2,	5,	exon,	
	ENST00000551420.1	2	3	5UTR		ENST00000434359.1,	2,	5,	5UTR,	
						ENST00000454289.2,	2,	6,	exon,	
						ENST00000457189.1,	2,	6,	5UTR,	
						ENST00000497617.1,	2,	7,	exon,	
						ENST00000540550.1,	2,	5,	exon,	
						ENST00000543727.1,	2,	6,	exon,	
						ENST00000548851.1,	2,	6,	exon,	
						ENST00000550559.1	2	7	exon	
**NDUFA13->YJEFN3**	ENST00000252576.5,	4,	5,	exon,	0	ENST00000397179.2,	4,	7,	exon,	0
	ENST00000502506.1,	4,	5,	exon,		ENST00000436027.2,	3,	6,	exon,	
	ENST00000503283.1,	4,	5,	exon,		ENST00000510139.1,	3,	6,	exon,	
	ENST00000507754.1,	4,	5,	exon,		ENST00000514277.1,	4,	7,	exon,	
	ENST00000512771.1	4	8	exon		ENST00000553705.1	5	7	exon	
**NDUFB8->SEC31B**	ENST00000299166.4,	4,	5,	exon,	0	ENST00000462434.1,	2,	25,	exon,	0
	ENST00000370320.4,	4,	5,	exon,		ENST00000535773.1	4	10	5UTR	
	ENST00000370322.1,	4,	5,	exon,						
	ENST00000464651.1,	1,	2,	exon,						
	ENST00000528425.1,	4,	5,	3UTR,						
	ENST00000531258.1	4	10	exon						
**NRXN1->EIF2AK2**	ENST00000342183.5	1	6	exon	176	ENST00000233057.4,	3,	17,	5UTR,	1
						ENST00000379156.2,	3,	13,	5UTR,	
						ENST00000390013.3,	3,	5,	5UTR,	
						ENST00000395127.2,	3,	17,	5UTR,	
						ENST00000411537.1	3	5	5UTR	
**NSUN4->FAAH**	ENST00000307089.3,	3,	5,	exon,	0	ENST00000243167.8,	2,	15,	exon,	0
	ENST00000471871.1,	4,	6,	exon,		ENST00000396325.4,	2,	10,	exon,	
	ENST00000474062.1,	4,	6,	exon,		ENST00000468718.1,	2,	5,	exon,	
	ENST00000474844.1,	4,	6,	exon,		ENST00000493735.1	2	8	exon	
	ENST00000486270.1,	4,	5,	exon,						
	ENST00000495427.1,	4,	6,	exon,						
	ENST00000498008.1,	4,	6,	exon,						
	ENST00000536062.1,	5,	7,	exon,						
	ENST00000537428.1	4	6	exon						
**PEX26->TUBA8**	ENST00000329627.5,	5,	6,	exon,	0	ENST00000316027.6,	2,	5,	5UTR,	0
	ENST00000399744.3,	4,	5,	exon,		ENST00000330423.3,	2,	5,	exon,	
	ENST00000399746.3	4	6	exon		ENST00000426208.1	2	3	5UTR	
**PFKFB4->SHISA5**	ENST00000232375.3,	13,	14,	exon,	0	ENST00000296444.2,	2,	6,	exon,	0
	ENST00000383734.2,	12,	13,	exon,		ENST00000415268.1,	3,	5,	3UTR,	
	ENST00000416568.1,	13,	14,	exon,		ENST00000417841.1,	3,	5,	5UTR,	
	ENST00000417753.1,	14,	15,	3UTR,		ENST00000417962.1,	3,	7,	3UTR,	
	ENST00000445633.1,	14,	15,	3UTR,		ENST00000424965.1,	2,	6,	exon,	

	ENST00000490115.1,	14,	15,	exon,		ENST00000441507.1,	4,	4,	3UTR,	
	ENST00000536104.1,	13,	14,	exon,		ENST00000442747.1,	2,	6,	5UTR,	
	ENST00000541519.1	14	15	exon		ENST00000443308.2,	2,	6,	exon,	
						ENST00000444115.1,	2,	6,	5UTR,	
						ENST00000490864.1	3	3	exon	
**PKHD1L1->EBAG9**	ENST00000378402.5,	76,	78,	exon,	0	ENST00000337573.5,	2,	7,	5UTR,	0
	ENST00000526472.1,	22,	24,	exon,		ENST00000395785.2,	2,	7,	5UTR,	
	ENST00000534623.1	2	3	exon		ENST00000527709.1,	2,	7,	5UTR,	
						ENST00000529502.1,	2,	6,	exon,	
						ENST00000530629.1	2	7	5UTR	
**PLEKHO2->ANKDD1A**	ENST00000323544.4,	5,	6,	exon,	0	ENST00000319580.8,	2,	6,	exon,	0
	ENST00000546008.1	5	7	exon		ENST00000357698.3,	4,	14,	exon,	
						ENST00000380230.3,	4,	15,	exon,	
						ENST00000395720.1,	4,	15,	exon,	
						ENST00000491145.1,	6,	10,	exon,	
						ENST00000496480.1,	2,	4,	exon,	
						ENST00000496660.1,	2,	6,	5UTR,	
						ENST00000513267.1	4	8	exon	
**POLA2->CDC42EP2**	ENST00000265465.3,	17,	18,	exon,	0	ENST00000279249.2,	2,	2,	5UTR,	0
	ENST00000525924.1,	8,	9,	exon,		ENST00000533419.1,	2,	2,	5UTR,	
	ENST00000527618.1,	10,	11,	exon,		ENST00000544348.1	2	2	5UTR	
	ENST00000534785.1	4	5	exon						
**PPRC1->NOLC1**	ENST00000278070.2,	13,	14,	exon,	0	ENST00000370007.4,	2,	13,	exon,	0
	ENST00000370012.1,	9,	10,	exon,		ENST00000405356.1,	2,	13,	exon,	
	ENST00000413464.2	11	12	exon		ENST00000464969.1,	2,	9,	exon,	
						ENST00000488254.1	2	12	exon	
**PRH1->PRR4**	ENST00000428168.2	4	5	3UTR	0	ENST00000228811.4,	2,	4,	exon,	0
						ENST00000431566.2,	2,	5,	exon,	
						ENST00000535024.1,	4,	6,	exon,	
						ENST00000536668.1,	9,	11,	exon,	
						ENST00000539179.1,	2,	3,	exon,	
						ENST00000540107.1,	2,	4,	exon,	
						ENST00000540808.1,	2,	3,	exon,	
						ENST00000544994.1	2	4	exon	
**PRIM1->NACA**	ENST00000338193.6,	12,	13,	exon,	0	ENST00000356769.3,	3,	8,	exon,	0
	ENST00000537418.1,	14,	15,	exon,		ENST00000393891.4,	3,	8,	exon,	
	ENST00000552590.1,	12,	13,	3UTR,		ENST00000454682.1,	4,	9,	exon,	
	ENST00000552853.1	11	12	exon		ENST00000546392.1,	3,	8,	exon,	
						ENST00000547914.1,	3,	7,	exon,	
						ENST00000549259.1,	3,	7,	exon,	
						ENST00000549855.1,	4,	6,	exon,	
						ENST00000550920.1,	2,	7,	exon,	
						ENST00000550952.1,	6,	11,	exon,	
						ENST00000551520.1,	3,	3,	exon,	
						ENST00000551775.1,	3,	6,	exon,	
						ENST00000551793.1,	3,	4,	exon,	
						ENST00000552540.1	3	8	exon	
**PRKAA1->TTC33**	ENST00000354209.3,	9,	10,	exon,	0	ENST00000337702.4,	2,	5,	exon,	0
	ENST00000397128.2	8	9	exon		ENST00000337702.4,	2,	5,	5UTR,	
						ENST00000503936.2,	2,	3,	exon,	
						ENST00000504251.2,	2,	4,	exon,	
						ENST00000511730.2	2	4	exon	
**PRR11->C17orf71**	ENST00000262293.3	9	10	exon	0	ENST00000300917.5,	2,	4,	exon,	0
						ENST00000543872.1	2	5	exon	
**PRR13->PCBP2**	ENST00000379786.4,	3,	4,	exon,	0	ENST00000359282.5,	2,	14,	5UTR,	0
	ENST00000429243.2,	3,	4,	exon,		ENST00000359462.5,	2,	15,	5UTR,	
	ENST00000546581.1,	3,	4,	exon,		ENST00000437231.1,	2,	13,	5UTR,	
	ENST00000547368.1,	3,	4,	exon,		ENST00000439930.2,	2,	15,	5UTR,	
	ENST00000549068.1,	3,	4,	3UTR,		ENST00000447282.1,	2,	14,	5UTR,	

	ENST00000549135.1,	3,	4,	exon,		ENST00000541275.1,	4,	13,	5UTR,	
	ENST00000549581.1,	3,	4,	exon,		ENST00000546463.1,	2,	15,	5UTR,	
	ENST00000549924.1,	3,	4,	exon,		ENST00000549863.1,	2,	13,	5UTR,	
	ENST00000551003.1	3	4	exon		ENST00000550192.1,	2,	5,	5UTR,	
						ENST00000550520.2,	2,	6,	5UTR,	
						ENST00000550927.1,	2,	12,	5UTR,	
						ENST00000551104.1,	2,	7,	5UTR,	
						ENST00000552083.2,	1,	13,	5UTR,	
						ENST00000552296.2	2	15	5UTR	
**PXMP2->PGAM5**	ENST00000317479.3,	2,	5,	exon,	0	ENST00000317555.2,	2,	6,	exon,	0
	ENST00000428960.2,	1,	5,	5UTR,		ENST00000498926.2,	2,	6,	exon,	
	ENST00000539093.1,	1,	3,	exon,		ENST00000543955.1	2	6	5UTR	
	ENST00000543589.1,	2,	3,	exon,						
	ENST00000545677.1	2	9	exon						
**RBM14->RBM4**	ENST00000310137.4,	1,	3,	exon,	0	ENST00000310092.7,	2,	4,	5UTR,	0
	ENST00000393979.3,	1,	3,	exon,		ENST00000396053.4,	2,	3,	5UTR,	
	ENST00000409372.1,	1,	3,	exon,		ENST00000398692.4,	2,	3,	5UTR,	
	ENST00000409738.4,	1,	2,	exon,		ENST00000408993.2,	2,	4,	5UTR,	
	ENST00000443702.1,	1,	3,	exon,		ENST00000483858.1,	2,	2,	5UTR,	
	ENST00000511114.1,	1,	2,	exon,		ENST00000506523.2,	2,	3,	5UTR,	
	ENST00000512283.1	1	2	exon		ENST00000510173.2,	2,	4,	5UTR,	
						ENST00000530235.1,	2,	3,	5UTR,	
						ENST00000532968.1	2	2	5UTR	
**RNASET2->RPS6KA2**	ENST00000028008.5,	6,	9,	3UTR,	0	ENST00000503859.1,	2,	22,	exon,	0
	ENST00000366855.6,	7,	10,	exon,		ENST00000506565.1,	3,	8,	exon,	
	ENST00000421787.1,	6,	9,	3UTR,		ENST00000510118.1	2	23	exon	
	ENST00000428859.2,	10,	13,	exon,						
	ENST00000467705.2,	3,	6,	exon,						
	ENST00000476238.2,	7,	10,	exon,						
	ENST00000478180.2,	7,	10,	exon,						
	ENST00000496851.2,	5,	7,	exon,						
	ENST00000499370.2,	5,	6,	exon,						
	ENST00000508775.1,	6,	9,	exon,						
	ENST00000509073.1	2	3	exon						
**RRM2->C2orf48**	ENST00000304567.4,	9,	10,	exon,	0	ENST00000381786.3	2	4	5UTR	0
	ENST00000360566.2,	9,	10,	exon,						
	ENST00000485717.1	4	5	exon						
**S1PR2->DNMT1**	ENST00000317726.3	1	2	5UTR	0	ENST00000340748.3,	2,	40,	exon,	0
						ENST00000359526.3,	2,	41,	exon,	
						ENST00000540357.1	2	40	exon	
**SDHAF2->C11orf66**	ENST00000301761.2,	3,	4,	exon,	0	ENST00000338608.2,	5,	14,	exon,	0
	ENST00000359614.5,	3,	5,	exon,		ENST00000432063.2	5	13	exon	
	ENST00000537782.1,	3,	5,	exon,						
	ENST00000542794.1	4	5	3UTR						
**SDHD->TEX12**	ENST00000375549.3,	3,	4,	exon,	0	ENST00000280358.4,	3,	5,	exon,	0
	ENST00000525291.1,	2,	3,	exon,		ENST00000530752.1	3	5	exon	
	ENST00000526592.1,	3,	5,	exon,						
	ENST00000528021.1,	3,	4,	exon,						
	ENST00000530923.1,	3,	5,	exon,						
	ENST00000534010.1	1	3	exon						
**SLC35A3->HIAT1**	ENST00000370153.1,	7,	8,	exon,	0	ENST00000370152.3	2	12	exon	0
	ENST00000370155.3,	7,	8,	exon,						
	ENST00000370156.3,	6,	7,	exon,						
	ENST00000427993.2,	8,	9,	exon,						
	ENST00000533028.1	7	9	exon						
**SLC39A1->CRTC2**	ENST00000310483.6,	4,	5,	exon,	0	ENST00000368633.1,	2,	14,	exon,	0
	ENST00000356205.4,	3,	4,	exon,		ENST00000476883.1,	2,	6,	exon,	
	ENST00000368621.1,	3,	4,	exon,		ENST00000487235.1,	2,	12,	exon,	
	ENST00000368623.3,	2,	3,	exon,		ENST00000492073.1	2	4	exon	

	ENST00000413622.1,	4,	5,	exon,						
	ENST00000417348.1,	3,	4,	exon,						
	ENST00000429040.1,	3,	4,	exon,						
	ENST00000543075.1	4	5	exon						
SLC43A3->PRG2	ENST00000352187.1,	13,	14,	exon,	0	ENST00000311862.5,	2,	6,	5UTR,	0
	ENST00000395123.2,	13,	14,	exon,		ENST00000525955.1,	2,	6,	5UTR,	
	ENST00000395124.1,	13,	14,	exon,		ENST00000530105.1,	2,	5,	exon,	
	ENST00000525205.1,	4,	5,	3UTR,		ENST00000533605.1	2	6	5UTR	
	ENST00000529554.1,	12,	13,	exon,						
	ENST00000533524.1	13	14	exon						
SMG1->ARL6IP1	ENST00000389467.3,	62,	63,	exon,	0	ENST00000304414.7,	2,	6,	exon,	0
	ENST00000446231.2,	62,	63,	exon,		ENST00000545430.1,	2,	7,	5UTR,	
	ENST00000565324.1	60	61	exon		ENST00000546206.2,	2,	6,	5UTR,	
						ENST00000562234.2,	2,	5,	exon,	
						ENST00000562819.1,	2,	3,	exon,	
						ENST00000563861.1,	2,	5,	exon,	
						ENST00000566391.1,	2,	2,	exon,	
						ENST00000567969.1,	2,	3,	exon,	
						ENST00000569976.1	2	4	exon	
SNTB2->VPS4A	ENST00000336278.4,	6,	7,	exon,	0	ENST00000254950.11,	2,	11,	exon,	0
	ENST00000467311.1	5	6	3UTR		ENST00000569775.1	2	6	exon	
SUMO2->HN1	ENST00000420826.1	3	4	exon	0	ENST00000304834.7,	3,	6,	3UTR,	0
						ENST00000356033.4,	2,	4,	exon,	
						ENST00000392566.2,	2,	5,	5UTR,	
						ENST00000392573.2,	3,	5,	3UTR,	
						ENST00000405458.3,	3,	6,	5UTR,	
						ENST00000409135.1,	2,	4,	exon,	
						ENST00000409753.3,	2,	5,	exon,	
						ENST00000465454.1,	2,	3,	exon,	
						ENST00000470924.1,	2,	5,	exon,	
						ENST00000476258.1,	2,	5,	exon,	
						ENST00000481647.1,	2,	5,	exon,	
						ENST00000482348.1	2	5	exon	
SYNJ2BP->COX16	ENST00000256366.4,	3,	4,	exon,	0	ENST00000389912.5	2	4	exon	0
	ENST00000554216.1	2	3	exon						
TAGLN2->CCDC19	ENST00000368097.4,	1,	5,	5UTR,	0	ENST00000368099.4,	2,	12,	exon,	0
	ENST00000478033.1	1	4	exon		ENST00000426543.2,	2,	12,	5UTR,	
						ENST00000476696.1,	2,	12,	exon,	
						ENST00000479940.1	3	7	exon	
TAP2->HLA-DOB	ENST00000374897.2,	11,	12,	exon,	0	ENST00000438763.2,	2,	6,	exon,	0
	ENST00000374899.4,	11,	12,	exon,		ENST00000447394.1,	2,	3,	exon,	
	ENST00000464100.1,	3,	4,	exon,		ENST00000475235.1	2	5	exon	
	ENST00000556934.1	11	15	exon						
TMBIM4->LLPH	ENST00000286424.7,	8,	8,	exon,	0	ENST00000266604.2,	2,	3,	5UTR,	1
	ENST00000358230.3,	7,	7,	exon,		ENST00000446587.2	2	3	5UTR	
	ENST00000539427.1,	11,	11,	exon,						
	ENST00000542724.1,	7,	7,	exon,						
	ENST00000544599.1	7	7	exon						
TNFAIP8L2->SCNM1	ENST00000368910.3	1	2	5UTR	0	ENST00000368902.1,	2,	7,	5UTR,	0
						ENST00000368905.4,	2,	7,	exon,	
						ENST00000461862.1,	2,	5,	exon,	
						ENST00000471039.1	2	2	exon	
TOMM5->FBXO10	ENST00000540941	2	4	3UTR	2	ENST00000276960.7,	2,	9,	5UTR,	0
						ENST00000432825.2,	2,	11,	5UTR,	
						ENST00000541607.1	3	3	exon	
TOPORS->DDX58	ENST00000360538.2	2	3	exon	0	ENST00000379868.1,	2,	17,	5UTR,	0
						ENST00000379883.2,	2,	18,	exon,	
						ENST00000542096.1,	2,	19,	5UTR,	
						ENST00000542960.1,	2,	7,	exon,	

						ENST00000545044.1	2	15	5UTR	
**TPD52L2->DNAJC5**	ENST00000217121.5,	3,	9,	exon,	0	ENST00000360864.4,	2,	5,	5UTR,	0
	ENST00000346249.4,	3,	7,	exon,		ENST00000369911.2,	2,	5,	5UTR,	
	ENST00000348257.5,	3,	6,	exon,		ENST00000470551.1	2	6	5UTR	
	ENST00000351424.4,	3,	8,	exon,						
	ENST00000352482.4,	3,	8,	exon,						
	ENST00000358548.4,	3,	7,	exon,						
	ENST00000369927.2	3	7	exon						
**TSC22D4->C7orf61**	ENST00000300181.2,	2,	5,	exon,	0	ENST00000332375.3	2	3	exon	0
	ENST00000393991.1,	2,	5,	exon,						
	ENST00000493217.1,	2,	3,	exon,						
	ENST00000496728.1	1	5	exon						
**TSTD1->F11R**	ENST00000318289.10,	1,	3,	exon,	0	ENST00000289779.3,	5,	13,	exon,	0
	ENST00000368023.3,	1,	4,	exon,		ENST00000335772.2,	4,	12,	exon,	
	ENST00000368024.1,	1,	3,	exon,		ENST00000368026.5,	2,	10,	exon,	
	ENST00000423014.2,	1,	4,	exon,		ENST00000436182.2,	2,	10,	exon,	
	ENST00000486084.1	1	2	exon		ENST00000470694.1,	4,	7,	exon,	
						ENST00000537746.1	2	9	exon	
**UBA2->WTIP**	ENST00000246548.3,	16,	17,	exon,	0	ENST00000270288.6	4	10	exon	0
	ENST00000439527.2	16	17	exon						
**UBE2J1->GABRR2**	ENST00000435041.2,	8,	8,	exon,	104,104	ENST00000402938.3	2	9	exon	0
	ENST00000536477.1	9	9	exon						
**UBE2J2->FAM132A**	ENST00000339385.6,	4,	6,	exon,	0	ENST00000330388.2	2	8	exon	0
	ENST00000347370.2,	5,	7,	exon,						
	ENST00000348298.7,	5,	7,	exon,						
	ENST00000349431.6,	5,	7,	exon,						
	ENST00000360466.2,	5,	7,	exon,						
	ENST00000400929.2,	4,	6,	exon,						
	ENST00000400930.4,	6,	8,	exon,						
	ENST00000435198.1,	5,	7,	exon,						
	ENST00000450390.2,	6,	8,	3UTR,						
	ENST00000464036.1,	6,	8,	3UTR,						
	ENST00000466752.1,	6,	6,	3UTR,						
	ENST00000473215.1,	5,	7,	3UTR,						
	ENST00000491779.1,	3,	4,	exon,						
	ENST00000509720.1	3	5	3UTR						
**UCHL3->LMO7**	ENST00000377589.1,	8,	10,	exon,	0	ENST00000341547.4,	1,	30,	exon,	69, 69, 69
	ENST00000377595.3,	7,	9,	exon,		ENST00000357063.3,	1,	31,	exon,	
	ENST00000419068.1,	5,	7,	exon		ENST00000377534.3	1	30	exon	
	ENST00000471792.1	6	6							
**UQCRQ->LEAP2**	ENST00000378665.1	2	2	3UTR	3	ENST00000296877.2,	2,	3,	exon,	0
						ENST00000485457.1	2	3	exon	
**VKORC1->PRSS53**	ENST00000300851.6,	2,	3,	exon,	0	ENST00000280606.6	1	11	exon	0
	ENST00000319788.7,	2,	4,	exon,						
	ENST00000394971.3,	2,	3,	exon,						
	ENST00000394975.2,	2,	3,	exon,						
	ENST00000472468.1,	1,	2,	5UTR,						
	ENST00000498155.1	2	3	exon						
**ZNF343->SNRPB**	ENST00000278772.4,	5,	6,	exon,	0	ENST00000303103.6,	2,	9,	exon,	0
	ENST00000358413.2,	6,	7,	exon,		ENST00000339610.6,	2,	7,	5UTR,	
	ENST00000381253.1,	5,	6,	exon,		ENST00000381342.2,	2,	7,	exon,	
	ENST00000421216.1,	5,	5,	exon,		ENST00000438552.2,	2,	7,	exon,	
	ENST00000445484.1,	6,	7,	exon,		ENST00000474384.1	2	8	exon	
	ENST00000465019.1	1	2	exon						

* the exact distances from the fusion breakpoint to the exon ends are shown only for exons having distances different from 0.

Table S7

Gene	Chimera	Position	SNP	Ref/Alt	MAF
**TAP2**	TAP2->HLA-DOB	32796144	rs241454	A->G	0.3
**RRM2**	RRM2->C2orf48	10270094	rs62129883	A->G	0.06
**PFKFB4**	PFKFB4->SHISA5	48556231	rs13094578	T->A	0.08
**JAK3**	JAK3->INSL3	17935626	rs11888	T->C	0.35
**IFRD1**	IFRD1->C7orf53	112116241	rs3183621	A->G	0.22
**CHURC1**	CHURC1->FNTB	65401695	rs6745	C->A	0.21
**CCL22**	CCL22->CX3CL1	57399240	rs72786863	T->C	0.01

Table S8

<table>
<tr><th>Gene</th><th>Chimera</th><th>Chr</th><th>Position</th><th>Variant</th><th>p_value</th><th>adjusted p-value</th><th>Reference / Alternative</th><th>Regulatory feature</th><th>Odd ratio</th><th>95 % CI</th><th>Intron Number</th><th>Transcript</th><th>Exon number</th><th>Position</th><th>RNA binding ptotein</th></tr>
<tr><td>GNG5</td><td>GNG5-> CTBS</td><td>1</td><td>84965623</td><td>rs56212819</td><td>1.37E-04</td><td>2.87E-03</td><td>C/T</td><td>NA</td><td>2.013343</td><td>1.33-3.04</td><td>2|2</td><td>ENST00000370641</td><td>2|3</td><td>downstream</td><td>HuR, PABPC1</td></tr>
<tr><td>GNG5</td><td>GNG5-> CTBS</td><td>1</td><td>84965364</td><td>rs12140555</td><td>2.25E-04</td><td>4.73E-03</td><td>C/T</td><td>NA</td><td>1.94752</td><td>1.29-2.94</td><td>3|3</td><td>ENST00000370641</td><td>2|3</td><td>downstream</td><td>HuR, PABPC1</td></tr>
<tr><td>GNG5</td><td>GNG5-> CTBS</td><td>1</td><td>84970426</td><td>rs56049713</td><td>2.25E-04</td><td>4.73E-03</td><td>C/CT</td><td>DNAseI, PolII, H2K4me3, H3K36me3, H3k27me3</td><td>1.94752</td><td>1.29-2.94</td><td>1|2</td><td>ENST00000370641</td><td>2|3</td><td>upstream</td><td>HuR, PABPC1</td></tr>
<tr><td>GNG5</td><td>GNG5-> CTBS</td><td>1</td><td>84970947</td><td>rs3813605</td><td>2.25E-04</td><td>4.73E-03</td><td>C/T</td><td>DNAseI, PolII, H2K4me3, H3K36me3, H3k27me3</td><td>1.94752</td><td>1.29-2.94</td><td>1|2</td><td>ENST00000370641</td><td>2|3</td><td>upstream</td><td>HuR, PABPC1</td></tr>
<tr><td>HMSD</td><td>HMSD-> SERPINB8</td><td>18</td><td>61620766</td><td>rs9945924</td><td>5.55E-17</td><td>8.66E-15</td><td>G/A</td><td>NA</td><td>4.79183</td><td>3.22-7.14</td><td>1|5</td><td>ENST00000481726.1</td><td>3|6</td><td>distant</td><td>NA</td></tr>
<tr><td>HMSD</td><td>HMSD-> SERPINB8</td><td>18</td><td>61622086</td><td>rs8090046</td><td>5.55E-17</td><td>8.66E-15</td><td>G/A</td><td>NA</td><td>4.79183</td><td>3.22-7.14</td><td>2|5</td><td>ENST00000481726.1</td><td>3|6</td><td>upstream</td><td>NA</td></tr>
<tr><td>HMSD</td><td>HMSD-> SERPINB8</td><td>18</td><td>61622216</td><td>rs8095151</td><td>5.55E-17</td><td>8.66E-15</td><td>A/T</td><td>NA</td><td>4.701968</td><td>3.16-7.00</td><td>2|5</td><td>ENST00000481726.1</td><td>3|6</td><td>upstream</td><td>NA</td></tr>
<tr><td>HMSD</td><td>HMSD-> SERPINB8</td><td>18</td><td>61623469</td><td>rs9944952</td><td>5.55E-17</td><td>8.66E-15</td><td>A/G</td><td>NA</td><td>4.701968</td><td>3.16-7.00</td><td>2|5</td><td>ENST00000481726.1</td><td>3|6</td><td>upstream</td><td>NA</td></tr>
<tr><td>HMSD</td><td>HMSD-> SERPINB8</td><td>18</td><td>61628150</td><td>rs9961020</td><td>1.11E-16</td><td>1.73E-14</td><td>T/C</td><td>NA</td><td>4.592593</td><td>3.09-6.83</td><td>3|5</td><td>ENST00000481726.1</td><td>3|6</td><td>downstream</td><td>NA</td></tr>
<tr><td>HMSD</td><td>HMSD-> SERPINB8</td><td>18</td><td>61628258</td><td>rs9963550</td><td>1.11E-16</td><td>1.73E-14</td><td>T/A</td><td>NA</td><td>4.592593</td><td>3.09-6.83</td><td>3|5</td><td>ENST00000481726.1</td><td>3|6</td><td>downstream</td><td>PABPC1</td></tr>
<tr><td>HMSD</td><td>HMSD-> SERPINB8</td><td>18</td><td>61634364</td><td>rs36210123</td><td>2.22E-16</td><td>3.46E-14</td><td>AGACTCTTT/A</td><td>NA</td><td>4.414716</td><td>2.97-6.56</td><td>3|5</td><td>ENST00000481726.1</td><td>3|6</td><td>downstream</td><td>NA</td></tr>
<tr><td>HMSD</td><td>HMSD-> SERPINB8</td><td>18</td><td>61619521</td><td>indel:1D_18_61619521</td><td>4.44E-16</td><td>6.93E-14</td><td>AT/A</td><td>NA</td><td>3.867857</td><td>2.60-5.75</td><td>1|5</td><td>ENST00000481726.1</td><td>3|6</td><td>distant</td><td>NA</td></tr>
<tr><td>HMSD</td><td>HMSD-> SERPINB8</td><td>18</td><td>61625358</td><td>rs9953837</td><td>5.55E-16</td><td>8.66E-14</td><td>T/C</td><td>NA</td><td>4.388787</td><td>2.96-6.51</td><td>2|5</td><td>ENST00000481726.1</td><td>3|6</td><td>upstream</td><td>NA</td></tr>
<tr><td>HMSD</td><td>HMSD-> SERPINB8</td><td>18</td><td>61631447</td><td>rs1006754</td><td>1.06E-15</td><td>1.65E-13</td><td>C/T</td><td>NA</td><td>4.36044</td><td>2.94-6.47</td><td>3|5</td><td>ENST00000481726.1</td><td>3|6</td><td>downstream</td><td>NA</td></tr>
<tr><td>HMSD</td><td>HMSD-> SERPINB8</td><td>18</td><td>61632704</td><td>rs55715438</td><td>1.06E-15</td><td>1.65E-13</td><td>A/G</td><td>NA</td><td>4.36044</td><td>2.94-6.47</td><td>3|5</td><td>ENST00000481726.1</td><td>3|6</td><td>downstream</td><td>NA</td></tr>
<tr><td>HMSD</td><td>HMSD-> SERPINB8</td><td>18</td><td>61633244</td><td>rs2009989</td><td>1.06E-15</td><td>1.65E-13</td><td>T/C</td><td>NA</td><td>4.36044</td><td>2.94-6.47</td><td>3|5</td><td>ENST00000481726.1</td><td>3|6</td><td>downstream</td><td>NA</td></tr>
<tr><td>HMSD</td><td>HMSD-> SERPINB8</td><td>18</td><td>61626118</td><td>rs9962574</td><td>1.19E-14</td><td>1.86E-12</td><td>G/T</td><td>NA</td><td>3.653963</td><td>2.44-5.48</td><td>2|5</td><td>ENST00000481726.1</td><td>3|6</td><td>upstream</td><td>NA</td></tr>
<tr><td>HMSD</td><td>HMSD-> SERPINB8</td><td>18</td><td>61619522</td><td>rs9957368</td><td>2.99E-14</td><td>4.67E-12</td><td>T/A</td><td>NA</td><td>3.415622</td><td>2.29-5.09</td><td>1|5</td><td>ENST00000481726.1</td><td>3|6</td><td>distant</td><td>NA</td></tr>
<tr><td>HMSD</td><td>HMSD-> SERPINB8</td><td>18</td><td>61624635</td><td>rs28478697</td><td>3.43E-14</td><td>5.35E-12</td><td>C/T</td><td>NA</td><td>3.554007</td><td>2.38-5.32</td><td>2|5</td><td>ENST00000481726.1</td><td>3|6</td><td>upstream</td><td>NA</td></tr>
<tr><td>HMSD</td><td>HMSD-> SERPINB8</td><td>18</td><td>61620037</td><td>rs9955410</td><td>8.97E-14</td><td>1.40E-11</td><td>A/C</td><td>NA</td><td>3.497024</td><td>2.33-5.24</td><td>1|5</td><td>ENST00000481726.1</td><td>3|6</td><td>distant</td><td>NA</td></tr>
<tr><td>HMSD</td><td>HMSD-> SERPINB8</td><td>18</td><td>61620179</td><td>rs9960367</td><td>8.97E-14</td><td>1.40E-11</td><td>T/C</td><td>NA</td><td>3.497024</td><td>2.33-5.24</td><td>1|5</td><td>ENST00000481726.1</td><td>3|6</td><td>distant</td><td>NA</td></tr>
<tr><td>HMSD</td><td>HMSD-> SERPINB8</td><td>18</td><td>61617950</td><td>rs201337479</td><td>1.63E-13</td><td>2.55E-11</td><td>AG/A</td><td>NA</td><td>3.347458</td><td>2.25-4.97</td><td>1|5</td><td>ENST00000481726.1</td><td>3|6</td><td>distant</td><td>NA</td></tr>
<tr><td>HMSD</td><td>HMSD-> SERPINB8</td><td>18</td><td>61617951</td><td>rs57236287</td><td>1.73E-13</td><td>2.70E-11</td><td>G/A</td><td>NA</td><td>3.397436</td><td>2.29-5.05</td><td>1|5</td><td>ENST00000481726.1</td><td>3|6</td><td>distant</td><td>NA</td></tr>
<tr><td>HMSD</td><td>HMSD-> SERPINB8</td><td>18</td><td>61622791</td><td>rs9952485</td><td>7.47E-13</td><td>1.17E-10</td><td>G/A</td><td>NA</td><td>3.491304</td><td>2.32-5.25</td><td>2|5</td><td>ENST00000481726.1</td><td>3|6</td><td>upstream</td><td>NA</td></tr>
<tr><td>HMSD</td><td>HMSD-> SERPINB8</td><td>18</td><td>61625396</td><td>rs8092289</td><td>1.17E-12</td><td>1.83E-10</td><td>C/T</td><td>NA</td><td>3.425</td><td>2.30-5.10</td><td>2|5</td><td>ENST00000481726.1</td><td>3|6</td><td>upstream</td><td>NA</td></tr>
<tr><td>HMSD</td><td>HMSD-> SERPINB8</td><td>18</td><td>61620406</td><td>rs66537233</td><td>3.05E-12</td><td>4.76E-10</td><td>T/C</td><td>NA</td><td>3.214362</td><td>2.17-4.77</td><td>1|5</td><td>ENST00000481726.1</td><td>3|6</td><td>distant</td><td>NA</td></tr>
<tr><td>HMSD</td><td>HMSD-> SERPINB8</td><td>18</td><td>61616690</td><td>rs7241250</td><td>4.97E-12</td><td>7.75E-10</td><td>T/C</td><td>NA</td><td>3.182615</td><td>2.14-4.74</td><td>1|5</td><td>ENST00000481726.1</td><td>3|6</td><td>distant</td><td>NA</td></tr>
<tr><td>HMSD</td><td>HMSD-> SERPINB8</td><td>18</td><td>61617949</td><td>indel:2D_18_61617949</td><td>5.72E-12</td><td>8.93E-10</td><td>AAG/A</td><td>NA</td><td>3.307266</td><td>2.24-4.89</td><td>3|5</td><td>ENST00000481726.1</td><td>3|6</td><td>downstream</td><td>NA</td></tr>
<tr><td>HMSD</td><td>HMSD-> SERPINB8</td><td>18</td><td>61629158</td><td>rs56390579</td><td>1.89E-11</td><td>2.95E-09</td><td>C/T</td><td>NA</td><td>3.182615</td><td>2.14-4.74</td><td>3|5</td><td>ENST00000481726.1</td><td>3|6</td><td>downstream</td><td>NA</td></tr>
<tr><td>HMSD</td><td>HMSD-> SERPINB8</td><td>18</td><td>61629397</td><td>rs9949621</td><td>1.89E-11</td><td>2.95E-09</td><td>T/G</td><td>NA</td><td>3.182615</td><td>2.14-4.74</td><td>3|5</td><td>ENST00000481726.1</td><td>3|6</td><td>downstream</td><td>NA</td></tr>
<tr><td>HMSD</td><td>HMSD-> SERPINB8</td><td>18</td><td>61630884</td><td>rs8090586</td><td>1.89E-11</td><td>2.95E-09</td><td>G/C</td><td>CTCF, DNAseI, PolII, H2K4me3, H3K36me3, H3k27me3</td><td>3.182615</td><td>2.14-4.74</td><td>3|5</td><td>ENST00000481726.1</td><td>3|6</td><td>downstream</td><td>NA</td></tr>
<tr><td>HMSD</td><td>HMSD-> SERPINB8</td><td>18</td><td>61631046</td><td>rs1006755</td><td>1.89E-11</td><td>2.95E-09</td><td>A/G</td><td>CTCF, DNAseI, PolII, H2K4me3, H3K36me3, H3k27me3</td><td>3.182615</td><td>2.14-4.74</td><td>3|5</td><td>ENST00000481726.1</td><td>3|6</td><td>downstream</td><td>NA</td></tr>
<tr><td>HMSD</td><td>HMSD-> SERPINB8</td><td>18</td><td>61630803</td><td>rs9950903</td><td>2.78E-11</td><td>4.34E-09</td><td>A/C</td><td>CTCF, DNAseI, PolII, H2K4me3, H3K36me3, H3k27me3</td><td>3.182615</td><td>2.14-4.74</td><td>3|5</td><td>ENST00000481726.1</td><td>3|6</td><td>downstream</td><td>NA</td></tr>
<tr><td>HMSD|SERPINB8</td><td>HMSD-> SERPINB8</td><td>18</td><td>61637225</td><td>rs6567407</td><td>2.85E-11</td><td>4.44E-09</td><td>A/G</td><td>DNAseI, PolII, H2K4me3, H3K36me3, H3k27me3</td><td>3.120962</td><td>2.11-4.63</td><td>3|5</td><td>ENST00000481726.1</td><td>3|6</td><td>downstream</td><td>NA</td></tr>
<tr><td>HMSD</td><td>HMSD-> SERPINB8</td><td>18</td><td>61630367</td><td>rs9961553</td><td>2.81E-10</td><td>4.44E-09</td><td>C/T</td><td>NA</td><td>3.659259</td><td>2.48-5.40</td><td>3|5</td><td>ENST00000481726.1</td><td>3|6</td><td>downstream</td><td>NA</td></tr>
<tr><td>HMSD</td><td>HMSD-> SERPINB8</td><td>18</td><td>61630474</td><td>rs199852339</td><td>1.50E-08</td><td>4.38E-08</td><td>C/CTTCT</td><td>NA</td><td>3.746556</td><td>2.37-5.93</td><td>3|5</td><td>ENST00000481726.1</td><td>3|6</td><td>downstream</td><td>NA</td></tr>
</table>

**HMSD**	HMSD-> SERPINB8	18	61626507	rs55700832	2.50E-08	2.35E-06	A/G	NA	2.687285	1.82-3.98	2|5	ENST00000481726.1	3|6	distant	NA
**HMSD**	HMSD-> SERPINB8	18	61617819	rs11152410	3.21E-08	3.90E-06	C/T	NA	2.548462	1.74-3.73	1|5	ENST00000481726.1	3|6	distant	NA
**HMSD**	HMSD-> SERPINB8	18	61633569	rs17072304	5.25E-08	5.01E-06	T/C	CTCF, DNAseI, PolII, H2K4me3, H3K36me3, H3k27me3	2.603878	1.77-3.84	3|5	ENST00000481726.1	3|6	downstream	NA
**HMSD**	HMSD-> SERPINB8	18	61631332	rs4510128	8.31E-08	8.19E-06	G/C	CTCF, DNAseI, PolII, H2K4me3, H3K36me3, H3k27me3	2.59625	1.76-3.83	3|5	ENST00000481726.1	3|6	downstream	NA
**HMSD**	HMSD-> SERPINB8	18	61633387	rs55963539	8.31E-08	1.30E-05	G/A	CTCF, DNAseI, PolII, H2K4me3, H3K36me3, H3k27me3	2.59625	1.76-3.83	3|5	ENST00000481726.1	3|6	downstream	NA
**HMSD**	HMSD-> SERPINB8	18	61617546	rs56033510	4.62E-07	1.30E-05	A/G	NA	2.332762	1.60-3.40	1|5	ENST00000481726.1	3|6	distant	NA
**HMSD**	HMSD-> SERPINB8	18	61630461	rs9960948	5.71E-06	7.20E-05	G/C	NA	3.3639	1.91-5.91	3|5	ENST00000481726.1	3|6	downstream	NA
**HMSD|SERPINB8**	HMSD-> SERPINB8	18	61637862	rs56321661	4.69E-10	8.91E-04	C/A	DNAseI, PolII, H2K4me3, H3K36me3, H3k27me3	3.087151	2.10-4.54	3|5, 1|6	ENST00000481726.1, ENST00000353706.2	3|6, 2|7	downstream |upstream	SLBP, PABPC1
**HMSD|SERPINB8**	HMSD-> SERPINB8	18	61637974	rs55928920	2.29E-07	3.58E-05	C/T	DNAseI, PolII, H2K4me3, H3K36me3, H3k27me3	2.549342	1.73-3.76	3|5, 1|6	ENST00000481726.1, ENST00000353706.2	3|6, 2|7	downstream |upstream	SLBP, PABPC1
**HMSD|SERPINB8**	HMSD-> SERPINB8	18	61637672	rs9961615	2.70E-06	4.21E-04	C/G	DNAseI, PolII, H2K4me3, H3K36me3, H3k27me3	2.313235	1.58-3.38	3|5, 1|6	ENST00000481726.1, ENST00000353706.2	3|6, 2|7	downstream |upstream	SLBP, PABPC1
**HMSD|SERPINB8**	HMSD-> SERPINB8	18	61639311	rs28374149	1.83E-05	2.86E-03	G/A	DNAseI, PolII, H2K4me3, H3K36me3, H3k27me3	2.315552	1.57-3.41	3|5, 1|6	ENST00000481726.1, ENST00000353706.2	3|6, 2|7	downstream |upstream	SLBP, PABPC1
**SERPINB8**	HMSD-> SERPINB8	18	61652035	rs6567409	4.46E-06	6.95E-04	G/A	NA	2.205569	1.44-3.37	5|6	ENST00000353706.2	2|7	distant	SLBP, PABPC1
**SERPINB8**	HMSD-> SERPINB8	18	61652113	indel:1I_18_61652113	2.34E-05	3.65E-03	T/TG		2.205538	1.44-3.38	5|6	ENST00000353706.2	2|7	distant	SLBP, PABPC1
**TAP2**	TAP2-> HLA-DOB	6	32796793	rs4148876	2.20E-16	4.77E-14	G/A	NA	18.748052	8.21-42.82	11|11	ENST00000374899.4	11|12	upstream	CELF1, SLBP, PABPC1
**TAP2**	TAP2-> HLA-DOB	6	32796856	rs201194354	2.20E-16	4.77E-14	GAC/G	NA	18.748052	8.21-42.82	11|11	ENST00000374899.4	11|12	upstream	CELF1, SLBP, PABPC1
**TAP2**	TAP2-> HLA-DOB	6	32796857	rs4148875	2.20E-16	4.77E-14	ACAC/A	NA	18.748052	8.21-42.82	11|11	ENST00000374899.4	11|12	upstream	CELF1, SLBP, PABPC1
**TAP2**	TAP2-> HLA-DOB	6	32786882	rs3763355	1.55E-11	3.37E-09	T/C	NA	8.530233	3.86-18.86	11|14	ENST00000556934.1	11|15	upstream	NA
**TAP2**	TAP2-> HLA-DOB	6	32786917	rs3763354	2.95E-06	6.41E-04	G/A	NA	0.218705	0.08-0.64	11|14	ENST00000556934.1	11|15	upstream	NA

Figure S1

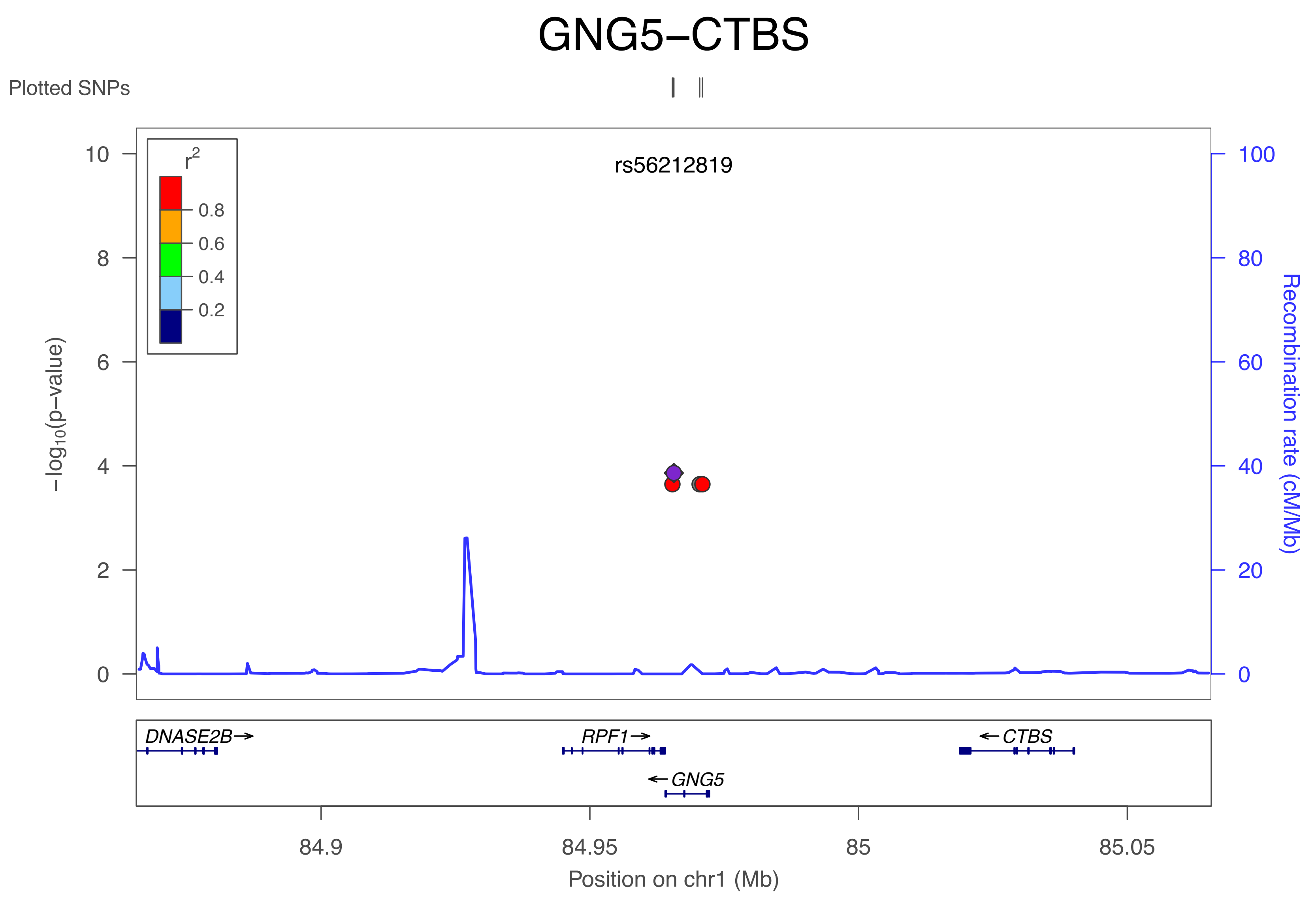

Figure S2

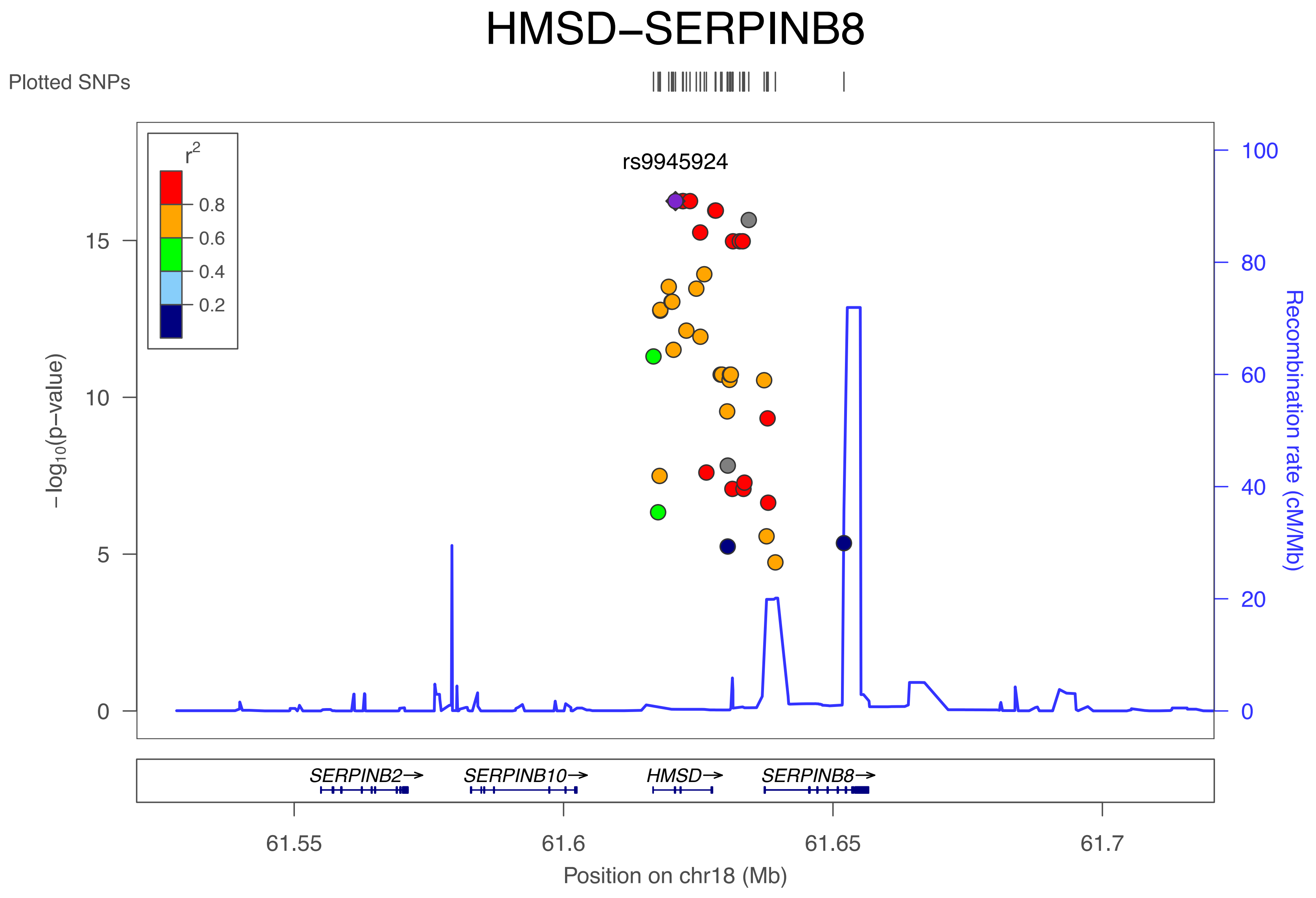

Figure S3

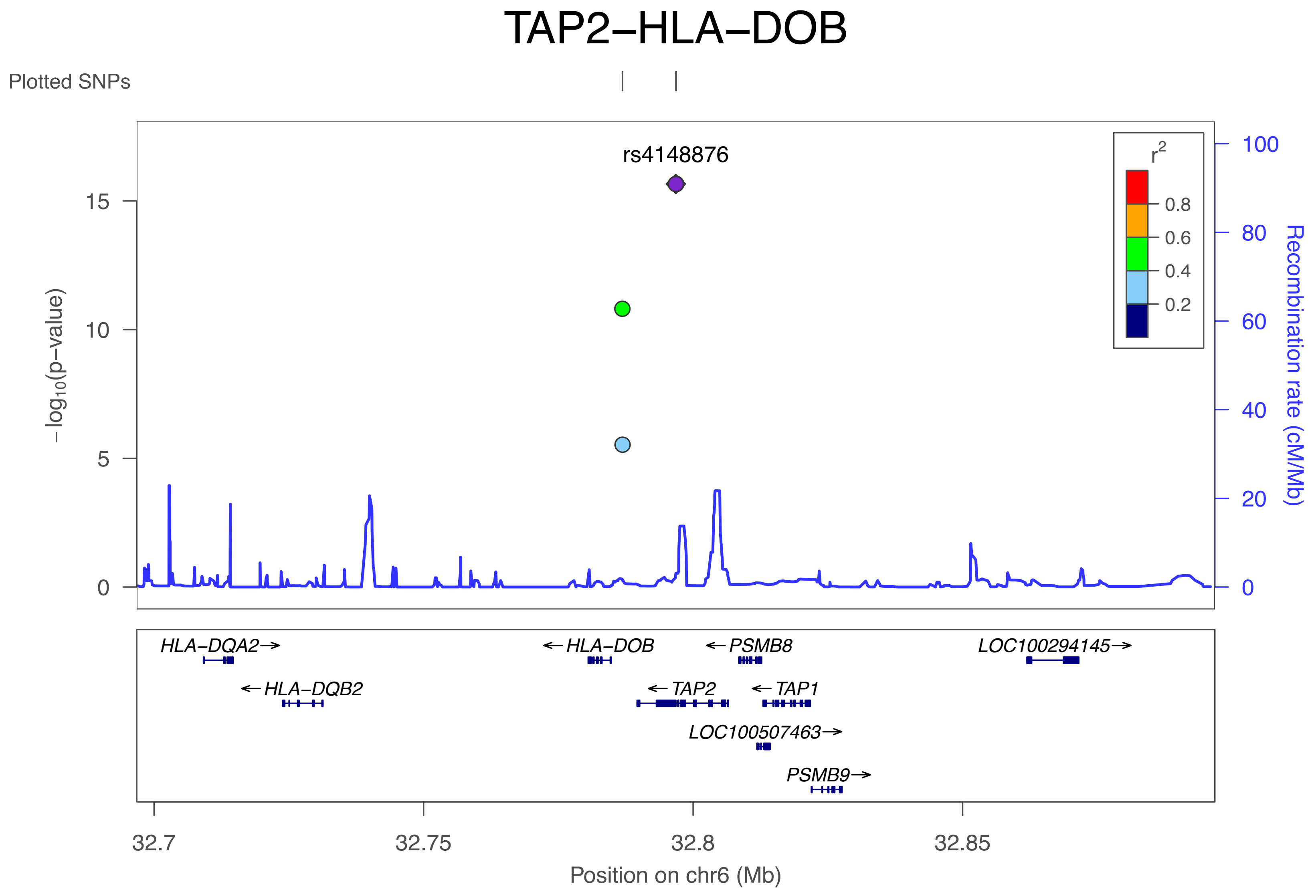
TAP2−HLA−DOB
Plotted SNPs
rs4148876
r2
0.8
0.6
0.4
0.2
100
80
60
40
20
0
Recombination rate (cM/Mb)
15
10
5
0
−log10(p−value)
HLA−DQA2
HLA−DQB2
HLA−DOB
PSMB8
TAP2
TAP1
LOC100507463
PSMB9
LOC100294145
32.7
32.75
32.8
32.85
Position on chr6 (Mb)
